# Systematic analysis of emotionality in consomic mouse strains established from C57BL/6J and wild-derived MSM/Ms

**DOI:** 10.1111/j.1601-183X.2008.00419.x

**Published:** 2008-11

**Authors:** A Takahashi, A Nishi, A Ishii, T Shiroishi, T Koide

**Affiliations:** † Mouse Genomics Resource Laboratory, National Institute of GeneticsMishima, Shizuoka; ‡ Department of Genetics, The Graduate University for Advanced Studies (SOKENDAI)Hayama; § Mammalian Genetics Laboratory, National Institute of GeneticsMishima, Shizuoka, Japan

**Keywords:** Emotionality, consomic mouse strains, wild-derived MSM, genetic mapping, QTL

## Abstract

Consomic strains have recently attracted attention as an advantageous method to screen for genes related to developmental, physiological, and behavioral phenotypes. Recently, a new set of consomic strains was established from the Japanese wild-derived mouse strain MSM/Ms and C57BL/6JJcl. By analyzing the entire consomic panel, we were able to identify a number of chromosomes associated with anxiety-like behaviors in the open-field (OF) test, a light–dark box and an elevated plus maze. Detailed observation of the OF behavior allowed us to identify chromosomes associated with those ethological traits, such as stretch attend, rearing, and jumping. Repeated OF test trials have different meanings for animals, and we found that some chromosomes responded to only the first or second trial, while others were consistent across both trials. By examining both male and female mice, sex-dependent effects were found in several measurements. Principal component analysis of anxiety-like behaviors extracted five factors: ‘general locomotor activity’, ‘thigmotaxis’, ‘risk assessment’, ‘open-arm exploration’ and ‘autonomic emotionality’. We mapped chromosomes associated with these five factors of emotionality.

Individual differences in most behaviors derive from polygenic influences, rather than Mendelian mutations with large effects (Plomin *et al.* 2001). To date, a vast number of quantitative trait loci (QTL) related to anxiety-like behaviors has been reported in mice and rats by using F2 intercross, N2 backcross, recombinant inbred strains, and heterogeneous stocks (Flint 2002, 2003; Valdar *et al.* 2006). Flint *et al.* (2005) reviewed several QTL studies and found that most QTL have just a small effect size, contributing approximately 6% of the total phenotypic variance for behavioral and physiological phenotypes. Also, extensive genome-wide high-resolution mapping using heterogeneous stock mice revealed 843 QTL for a variety of phenotypes, including behavior, and found that only 10 QTL had effect sizes of greater than 5%, while 109 QTL had less than 2% (Valdar *et al.* 2006). Because of this small effect of each QTL, an enormous amount of effort is required to identify quantitative trait genes (QTGs) for behavior.

Consomic strains, also known as chromosome substitution strains, are a favorable resources for investigating QTG; genotyping to map the chromosome is unnecessary, results are reproducible, QTL detection is statistically significant, and making congenic strains is rapid (Belknap 2003; Nadeau *et al.* 2000). Analysis of consomic strains established from C57BL/6J (B6) and A/J has successfully shown the chromosomes affecting several phenotypes including anxiety-related behaviors (Laarakker *et al.* 2008; Ponder *et al.* 2007; Singer *et al.* 2004, 2005). Recently, a new set of consomic strains was established, dubbed B6-ChrN^MSM^ consomic panel mice, using a different subspecies group of mouse strain MSM/Ms (MSM) (Takada *et al.* 2008). In this panel, each of the MSM chromosomes was introduced into the B6 background to encompass the whole genome. MSM was derived from Japanese wild mice (*Mus musculus molossinus*), and they had not undergone a strong selection history for domestication during breeding. Thus, it was expected that they would retain several behavioral characteristics of wild mice. It is known that several behavioral responses have been changed or sometimes attenuated in standard laboratory strains (Blanchard *et al.* 1998; Fernandes *et al.* 2004; Holmes *et al.* 2000; Koide *et al.* 2000), and thus, wild-derived mouse strains may offer interesting alternatives for behavioral analysis. We previously showed that MSM exhibited higher spontaneous activity in the home cage, reduced novelty-induced activity and increased freezing and grooming in a novel situation, difficulty in habituation to novelty, and reduced pain sensitivity compared to B6 (Koide *et al.* 2000; Takahashi *et al.* 2006). Consomic strains derived from MSM are expected to be useful for identifying genetic loci associated with the widely diverse phenotypes, some of which may have been lost in the laboratory strains. To date, QTL associated with hybrid sterility have been mapped on chromosome X (Oka *et al.* 2004, 2007), and a resistant gene for age-related hearing loss in B6 was mapped on chromosome 17 (Nemoto *et al.* 2004) by using B6-ChrN^MSM^ consomic panel mice. However, no systematic behavioral characterization has been done in these consomic strains.

Here, we performed behavioral characterizations for B6-ChrN^MSM^ consomic panel mice for anxiety-like behaviors [open-field (OF) test, light–dark (LD) box, and elevated plus maze (EPM)] to map the chromosomes associated with those behaviors. In this study, both males and females were separately analyzed to examine sex differences. Multivariate analysis was performed on anxiety-like behaviors to examine genetic relations among behaviors and to map the chromosomes related to fundamental constructs that underlie emotionality.

## Materials and methods

### Animals

**Figure 1:**
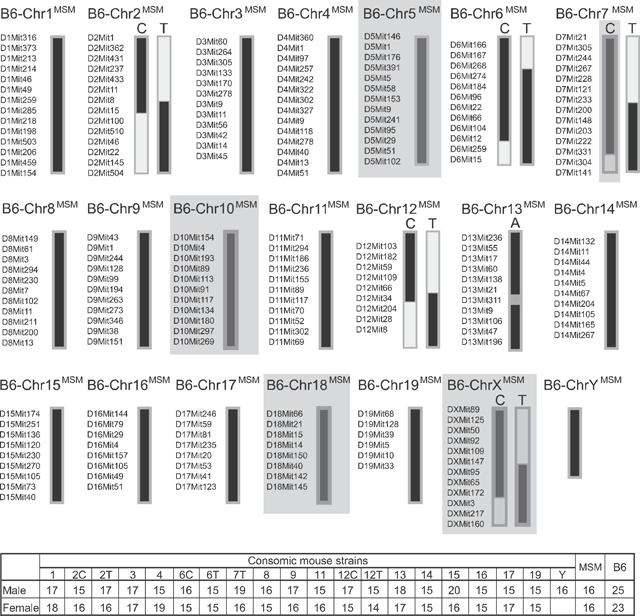
A panel of consomic strains established from C57BL/6J (B6) and MSM/Ms (MSM) MIT microsatellite markers used to establish each consomic strain are listed. Note that the B6-Chr13A^MSM^ strain possesses a heterozygote locus around the D13Mit311 region. Strains that did not complete the behavioral characterization are in gray. The lower table indicates the number of animals used in this study for each consomic strain and the parental strain.

MSM/Ms (abbreviated as MSM) was established as an inbred strain after 20 generations of brother–sister mating at the National Institute of Genetics (NIG; Mishima, Japan), and C57BL/6JJcl (abbreviated as B6) was purchased from CLEA Japan, Inc (Tokyo, Japan) and bred at NIG. Figure 1 shows the panel of consomic strains used in this study. Establishment of the B6-ChrN^MSM^ consomic panel has been described in detail by Takada *et al.* (2008). Briefly, MSM was backcrossed to B6 for more than 10 generations. In each generation, genotyping was performed by using MIT markers distributed on the desired chromosome (Fig. 1). All consomic strains had the same genetic background as B6, except for one pair of chromosomes, which were replaced with the corresponding MSM chromosomes. It proved difficult to substitute the whole chromosome for chromosomes 2, 6, 7, and 12, and, accordingly, two subconsomic strains were established for each of those chromosomes to cover the whole chromosome [telomeric (T) and centromeric (C); see Fig. 1]. Behavioral characterization of some consomic strains was not completed because of the poor breeding performance of the strains (Fig. 1 gray color). Each consomic strain was dubbed B6-ChrN^MSM^, where N is the chromosome number transferred from the MSM strain. All animals were maintained at NIG under a 12:12 h light : dark cycle (light from 800 to 2000 h) in a temperature-controlled room (23 ± 2°C). The mice were weaned around 3–4 weeks of age and housed in same sex groups in standard size plastic cages on wood chips. Before starting the behavioral tests, each mouse was housed in a single cage for about 7 days to measure home cage activity, and they were singly housed continuously for the duration of testing. Food and water were available *ad libitum*. Mice were maintained according to NIG guidelines, and all procedures were carried out with approval from our institutional animal care and use committee.

### Behavioral testing

A battery of tests for behavioral characterization of the consomic panel was conducted in the animal facility in which mice were bred. This behavioral battery started at the age of 10–12 weeks, and ended at 12–15 weeks old. All tests were carried out during the light period (1300–2000 h). Every mouse experienced a series of behavioral tests in the same order with at least one day's rest between consecutive tests (tests were performed in the following order). The number of animals used in this study is indicated in Fig. 1 [the number of animals analyzed was reduced (by up to three) in some strains for some tests because of computer data capture failure, but all animals underwent the same tests in the same order].

#### Open-field test

Open-field tests were conducted based on a previous report (Takahashi *et al.* 2006). Open-field used consisted of a square arena (60 × 60 × 40 cm) made of a white polyvinyl chloride plastic board divided into 16 equal squares. The arena was brightly lit by incandescent lighting (365 lux). During the 10-min trial, we observed their behavior directly and scored the presence or absence of 11 behavioral items (sniffing, locomotion, stretch-attend posture (stretch attend), leaning against a wall (leaning), rearing, grooming, face washing, digging, gnawing, jumping, pausing, and freezing) in each 5-second period in real time by a well-trained observer. Details of each item are summarized in Table S1.

To analyze ambulation (number of square transits), central ambulation, percentage of central ambulation, and time spent in the center, the arena was continuously recorded by a video camera placed over its center and relayed to a video tracking system (Image OF; O‘hara & Co., Ltd., Tokyo, Japan), which was based on National Institutes of Health (NIH) image. At the end of the test, the number of fecal boli (defecations) was recorded. Two tests were administered on two consecutive days.

#### Light–dark box test

The apparatus, SCANET MV-10 and SCANET MV-20 (Melquest Co., Ltd., Toyama, Japan), consisted of coupled black and transparent acrylic chambers (each measuring 15 × 15 × 16 cm) separated by a black acrylic board with an aperture of 4 cm in diameter between them. To start the measurement, mice were placed individually into the light chamber (95 lux). Then, the latency of the first transition into the dark chamber (0 lux), number of transitions between LD chambers and time spent in the dark chamber were measured for 10 min.

#### Elevated plus maze test

The apparatus, made of a white acrylic board, consisted of two open arms with low edges (30 × 5 × 0.25 cm) and two closed arms enclosed by a clear acrylic plastic wall (30 × 5 × 15 cm) that extended from a central platform (5 × 5 cm). It was elevated 60 cm above the floor and was dimly lit (150 lux). Mice were placed individually in the center platform and allowed to move freely for 10 min. Ambulatory activity (cm), number of entries into the open arm or closed arm and duration in the open arm or closed arm were measured by a video tracking system (Image EPM; O'hara & Co. Ltd.), which was based on NIH image.

### Statistical analysis

Data analysis was performed using the spssversion 14.0J software package (SPSS Inc., Chicago, IL, USA). In order to avoid the interactive influence of sex chromosomes, males and females were separately analyzed in this study. One-way anovawas performed to examine the effect of strain, and then, the significance of each consomic strain compared to B6 was determined by a *t*-test with a Bonferroni correction (*p* =*α*/*m*, where *α* = 0.05 and *m* = 20 for males and *m* = 19 for females). To examine sex–genotype interaction, a two-way anovawas performed in all consomic strains and B6. Some behavioral items were excluded from this analysis because of the floor effect (gnawing, digging and freezing) or ceiling effect (sniffing). The OF test was performed twice, and repeated measures one-way anovawas conducted to examine the trial–genotype interaction of each sex individually.

### Principal component analysis

Multivariate analysis was performed using the SPSS version 14.0J software package. First, Pearson's correlations were calculated for phenotypic correlations (calculated using individual values) and approximate genetic correlations (calculated using the mean score for each strain) in all consomic strains and B6 (Blizard & Bailey 1979; Crusio 2007). Approximate genetic correlations were estimated using mean scores of both males and females of each strain because there are significant sex effects for some variables. Principal component analysis with oblique rotation was performed to reveal both phenotypic and genetic correlations. An eigenvalue greater than 1 was used as the criterion for selecting factors. Factor scores for individual animals were estimated by summing each value that was weighted with the eigen vector of each factor. These factor scores were subjected to one-way anovato examine the effect of strain, and then exposed to a *t*-test with a Bonferroni correction to compare them with B6 (*p* =*α*/*m*, where *α* = 0.05 and *m* = 20 for males and *m* = 19 for females). To examine sex–genotype interaction, two-way anovawas performed in all consomic strains and B6.

## Results

### Mapping the chromosomes associated with anxiety-like behaviors

The Student's *t*-test revealed that the parental B6 and MSM strains showed substantially different behavioral patterns in the OF test; all indices except leaning and jumping (and face washing in males) showed significant differences in both males and females. In LD, females showed reduced transit and increased dark box duration, but males did not show any differences compared to corresponding sex of B6. In EPM, there were significant differences in activity measurements but not in open-arm exploration indices in both males and females.

We then identified the chromosomes associated with anxiety-like behaviors. One-way anovarevealed a significant effect of strain in all 35 behavioral measurements in both males (*F*_20,338–353_ > 1.880, *P* < .02) and females (*F*_19,317–330_ > 2.337, *P* < .002). A *t*-test with Bonferroni correction revealed that consomic strains showing significant differences from the host strain B6 in these 35 variables numbered 107 for males and 146 for females (male vs. female frequency, χ^2^(1) = 10.65, *P* < .01, Table 1), and 54% of them were common chromosomes in males and females. Two-way anovarevealed significant sex–genotype interaction for the first OF ambulation, percentage of central ambulation, EPM total arm entry, and closed-arm entry (*P* < .01), and first OF center ambulation, stretch attend, second OF ambulation, locomotion, leaning, grooming, and EPM total distance (*P* < .05). To evaluate the magnitude of sex–genotype interaction, we estimated *η*^2^ effect sizes for genotype, sex, and sex–genotype interaction (Table S2). The *η*^2^ estimates revealed that the magnitude of the sex–genotype interaction was smaller than the genotype effect, even in the measurements that had significant sex–genotype interaction. The differences in each behavior are shown in Figs S1–S4.

**Table 1:** Chromosomal mapping for anxiety-like behaviors using consomic mouse strains established from C57BL/6J (B6) and MSM/Ms (MSM)

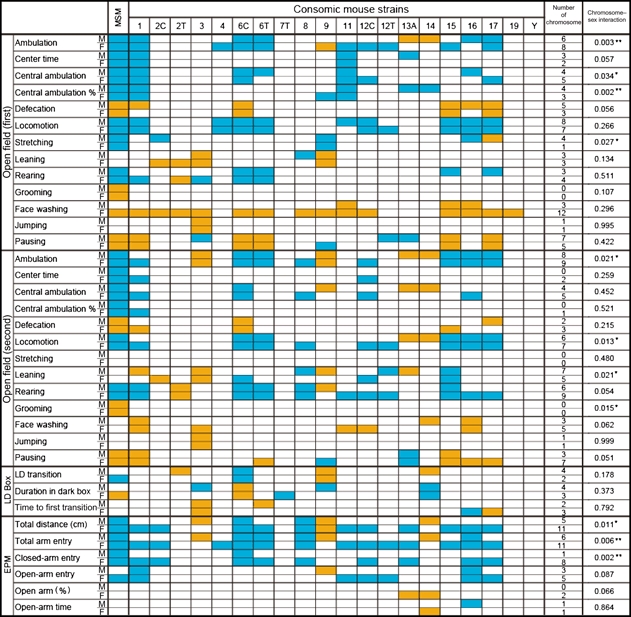

M, male; F, female. Colored cells indicate the consomic strains that showed significant differences from B6 (*P* < .05 with a Bonferroni correction). Orange: significant increase; blue: significant decrease from corresponding sex of B6. . Number of chromosomes associated with the behavior was estimated for each measurement. Chromosome–sex interaction was also estimated by two-way anovafor strain and sex

(** *P* < .01,

* *P* < .05).

For most variables, multiple consomic strains showed significant differences from B6. Activity in OF and EPM tended to be influenced by many chromosomes. In contrast, we failed to find any chromosome related to grooming, which is a prominent feature of MSM behavior (Takahashi *et al.* 2006). Jumping was especially characteristic in one strain, B6-Chr3^MSM^, while both parental strains showed only low levels of this behavior. For all measurements, B6-ChrY^MSM^ did not show any differences from B6.

We conducted repeated OF tests and found that the behaviors in the first and second trials of the OF tests were very different. Repeated measures one-way anovarevealed significant trial–genotype interaction in all OF measurements (*F*_20,660_ > 2.28, *P* < .05 with a Bonferroni correction) except grooming. Several chromosomes were associated with either the first or second trial, but some others showed significant effects in both trials. Those consomic strains were divided into three categories (Table 2); chromosomes that exhibited the first-trial-specific difference (26 and 20 consomic strains in males and females, respectively), second-trial-specific difference (16 and 17 strains) and differences in both trials (18 and 30 strains). Stretch attend and percentage of central ambulation were mostly affected in the first-trial-specific QTL but not by common QTL or second-trial-specific QTL. In contrast, rearing was affected mainly by second-trial-specific QTL. Ambulation and jumping showed effects mostly from common QTL.

**Table 2:** Three types of QTL that responded differentially in repeated OF trials

	Common QTL		First-trial-specific QTL		Second-trial-specific QTL	
			
	Male	Female	Male	Female	Male	Female
Ambulation	5	7	1	2	3	2
Center time	0	1	3	1	0	1
Central ambulation	1	3	4	2	3	2
Central ambulation %	0	1	4	2	0	0
Defecation	2	2	3	1	0	1
Locomotion	4	6	4	1	2	1
Stretch attend	0	0	4	1	0	0
Leaning	3	2	0	2	4	3
Rearing	2	3	1	1	4	6
Grooming	0	0	0	0	0	0
Face washing	1	5	2	7	2	0
Jumping	1	1	0	0	0	0
Pausing	3	5	4	1	0	2

Common QTL: the number of consomic strains that showed significant differences for both trials in the same direction. First-trial-specific QTL: the number of consomic strains that exhibited significant differences only in the first trial. Second-trial-specific QTL: the number of consomic strains that showed significant differences only in the second trial.

### Multivariate analysis of anxiety-like behaviors in consomic strains

Table S3 shows phenotypic and approximate genetic correlations between each of the 37 measurements for anxiety-like traits in 20 consomic strains and B6. To extract some general factors underlying anxiety-like behaviors, principal component analysis was performed on all measurements in this study, including the first trial of OF, LD and EPM. As OF jumping was very skewed and leptokurtic because of a much higher value of B6-Chr3^MSM^, this index seemed to affect the factor structure inappropriately and was excluded from this analysis. A single measurement was used when two or more variables were considered as similar and highly correlated [*r* > 0.90; e.g. OF ambulation (number of square crossing) and locomotion (5-second time sampling), *r* = 0.92].

We first performed principal component analysis with oblique rotation on the phenotypic correlation matrix and found that these factors were relatively test specific (Table S4). In contrast, principal component analysis of the strain correlation matrix extracted factors that tended to have more cross-test contributions compared to the phenotypic principal component analysis. Five factors accounting for 79.1% of total variance with eigenvalues greater than 1 were extracted by principal component analyses with oblique rotation (Table 3). Factor 1 had positive loadings from OF ambulation, LD transition and EPM arm entry, and negative loadings from LD dark box preference. Thus, this first factor was named as ‘general locomotor activity’. Factor 2 had positive loadings from OF leaning and face washing and negative loadings from OF center activity and grooming. Because of opposite loadings of OF center activity measurements and leaning, which occurs beside a wall, this factor was named ‘thigmotaxis’ (Takahashi *et al.* 2006; Treit & Fundytus 1989). Factor 3 was named ‘EPM open-arm exploration’ because it had positive loadings from EPM open-arm exploration measurements. Factor 4 had positive loadings from OF stretch attend and LD hesitancy of first transit but negative loadings for OF rearing. Therefore, factor 4 was named as ‘risk assessment’, which occurs in approach-avoid conflict situations (Blanchard *et al.* 1991a; Carola *et al.* 2002; Rodgers & Johnson 1995; Takahashi *et al.* 2006). Factor 5 had positive loadings from OF defecation and pausing and was named as ‘autonomic emotionality’ because defecation has long been considered an autonomic response of emotionality (Hall 1934). There was a moderate correlation between factors 1 and 5 (*r* = −0.45) but no, or quite a weak, correlation among other factors.

**Table 3:** Principal component analysis with oblique rotation for emotionality-related tests of the strain correlation matrix

	1	2	3	4	5
Open field					
Ambulation	**0.50**	0.15	0.32	−0.05	−0.47
Center ambulation	0.39	−**0.50**	0.05	0.05	−**0.51**
Center %	−0.01	−**0.81**	−0.28	0.17	−0.05
Defecation	−0.01	−0.06	0.21	−0.01	**0.89**
Stretch attend	−0.49	−0.06	0.13	**0.64**	−0.14
Leaning	**0.53**	**0.64**	0.04	0.12	−0.05
Rearing	0.39	−0.35	−0.09	−**0.51**	−0.30
Grooming	−0.07	−**0.62**	0.03	−0.07	0.46
Face washing	−0.10	**0.53**	−0.19	−0.46	0.18
Pausing	−0.02	0.05	−0.17	0.21	**0.86**
LD box					
Transition	**0.84**	0.10	−0.04	−0.19	0.01
First transit latency	0.16	−0.07	−0.09	**0.91**	0.17
Dark box duration	−**0.84**	0.11	−0.10	−0.07	−0.01
EPM					
Total arm entry	**0.68**	0.15	0.05	0.24	−0.34
Open-arm entry %	0.01	0.04	**0.93**	−0.04	0.23
Open-arm time	0.01	0.03	**0.92**	0.03	−0.14

Factor loadings more than 0.5 are given in boldface.

### Representation of each consomic strain in terms of the five factors

To identify chromosomes that contributed to the five factors, we next calculated factor scores for each individual using factor loadings extracted from genetic principal component analysis. Because the number of variables analyzed in Table 1 was high, there was a statistical problem of multiple comparisons, even with a corrected *P* value, with the number of strains. Principal component analysis is also useful for reducing the number of variables to increase the statistical power and identify chromosomes with lower false positives. Table 4 and Fig. S5 show consomic strains related to each factor. One-way anovarevealed significant effects of strain in all factors in males (*F*_20, 352_ ≤ 3.464, *P* < .001) and females (*F*_19,330_ ≤ 6.191, *P* < .001). Factor 1, general locomotor activity, increased in both sexes of B6-Chr9^MSM^ and males of consomic strains for chromosomes 3 and 14 but decreased in both sexes of B6-Chr6C^MSM^ and females of consomic strains for chromosomes 1 and 12C. Factor 2, thigmotaxis, showed contributions from both sexes of consomic strains for chromosomes 1 and 11, males of chromosome 13 and females of chromosomes 3 and 9. Factor 3, EPM open-arm exploration, increased in females of consomic strains for chromosomes 13 and 14, whereas reduced in males of chromosome 16. For factor 4, risk assessment, both sexes of B6-Chr3^MSM^ and males of consomic strains for chromosomes 6C and 17 showed a significant increase, whereas both sexes of consomic strains for chromosomes 9 and 16 and females of consomic strains for chromosomes 2T and 11 showed a significant decrease compared to B6. Factor 5, autonomic emotionality, increased in both sexes of consomic strains for chromosomes 1, 2C, 6, 12C, 15, 16 and 17 and females of chromosomes 2T and 11. However, two-way anovarevealed significant sex–genotype interaction only for factor 2 (*F*_19,643_ = 2.251, *P* = .002). We did not find any statistically significant effect of chromosomes 4, 7T, 8, 12T, 19 and Y for any of these five factors.

**Table 4:** Chromosomal mapping for five factors extracted from principal component analysis

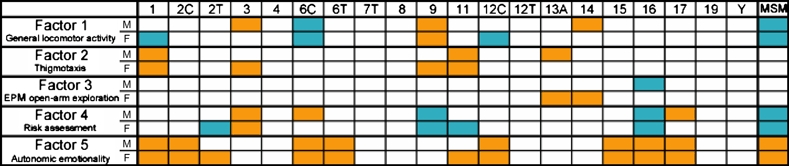

Orange: significant increase compared to B6; blue: significant decrease compared to B6 (*P* < .05 with a Bonferroni correction).

## Discussion

### Mapping the chromosomes associated with anxiety-like behaviors

In this study, we performed chromosomal mapping of anxiety-like behaviors by using a series of consomic strains established from B6 and MSM (B6-ChrN^MSM^). The wild-derived strain MSM belongs to *M. m. molossinus*, while most laboratory mouse strains were derived from the same small original population belonging largely to *M. m. domesticus*(Bonhomme & Guénet 1996; Ferris *et al.* 1982; Wade *et al.* 2002, Yonekawa *et al.* 1982). The rate of overall nucleotide substitution between B6 and MSM was estimated to be around 0.0096 (Abe *et al.* 2006), much more polymorphic than usual among laboratory strains. Therefore, B6-ChrN^MSM^ consomic panel mice were expected to provide a higher QTL yield than consomic sets derived from domesticus stocks.

Our results indicated that there are a large number of chromosomes related to three kinds of emotionality-related tests. Further analyses of some of these consomic strains revealed that each chromosome contains more than one QTL (in preparation). Several consomic strains showed a strong effect on the phenotype, and even one chromosome substitution caused the same or larger phenotype differences than parental MSM. Our results are comparable to those of another group using males of B6-A/J consomic strains for OF and LD tests (Singer *et al.* 2004, 2005). Interestingly, we found that a larger number of chromosomes in the B6-ChrN^MSM^ consomic panel associated with each behavior than the B6-A/J consomic panel. For example, Singer et al. (2005) reported that males of B6-Chr11^A/J^ showed center avoidance in OF. Our study using a B6-ChrN^MSM^ consomic panel revealed that chromosomes 1, 13 and 14, as well as 11, related to center avoidance in males. Among them, consomic strains for chromosomes 13 and 14 exhibited increased OF ambulation; thus, they were considered to have high thigmotaxis locomotor activity. The differences in the results between the B6-A/J consomic panel and our B6-MSM consomic panel may have been caused by the larger genetic distance between MSM and B6 than between A/J and B6. However, we cannot ignore the methodological differences (e.g. test length) between the two studies. Also, differences in statistical power have to be considered because the sample size in our study was 15–20 mice for each sex from 20 strains, which is a larger number of animals from fewer strains than in the B6-A/J consomic panel (Singer *et al.* 2005), leading to higher statistical power in our study.

On the one hand, we could not find chromosomes related to some behaviors that are characteristic in MSM: grooming and freezing in OF (Takahashi *et al.* 2006). It has been reported that freezing is not observed in commonly used laboratory mice but occurs frequently in wild mice (Blanchard *et al.* 1998; Fernandes *et al.* 2004; Holmes *et al.* 2000), and thus B6-ChrN^MSM^ consomic panel mice were expected to be an interesting tool to identify genetic loci associated with ‘wildness’-related phenotypes. However, our results suggested that freezing and grooming are multigenic traits that do not show up on a predominantly B6 background. On the other hand, we identified a chromosome related to jumping behavior that is also reported as a characteristic behavior of wild-derived strains (Fernandes *et al.* 2004; Holmes *et al.* 2000; Takahashi *et al.* 2006). Interestingly, although neither B6 nor MSM showed this behavior, consomic strains having chromosome 3 of MSM showed a high frequency of jumping even compared to other wild-derived strains (Takahashi *et al.* 2006). This result suggested that MSM retains genetic locus/loci that increase jumping as in other wild mouse strains, while it also possessed inhibitory loci for jumping behavior in novel situations. As a result, MSM did not exhibit jumping behavior.

### A number of sex-dependent QTL in the consomic strains

In this study, we analyzed males and females separately to consider sex differences. Our data suggested that there were many chromosomes that tended to have a prominent effect in either males or females, named as sex-dependent QTL. Two thirds of significant consomic strains showed sex-dependent differences, and females tended to have more chromosomes with significant effects than males. A statistically significant sex–genotype interaction was found in some behavioral measurements, and thus, several consomic strains for those indices may have QTL that have a sex-‘specific’ effect on these phenotypes. However, from the *η*^2^ estimates, the effects of these sex-specific QTL are moderate. It has been reported that there are sex differences and sex–genotype interaction in anxiety-like behavior (Blanchard *et al.* 1991b, Holmes *et al.* 2000; Ramos *et al.* 1999), and it was also reported that the quality of anxiety-like behavior varied between males and females; male behaviors are driven by sexual preference and anxiety, while female behaviors are characterized primarily by motor activity in rats (Fernandes *et al.* 1999). The loci we found in this study may be related to the ‘quality’ of emotionality. These sex-specific effects might be because of the epistatic effects of sex chromosomes and some other sexual dimorphic genes (Yang *et al.* 2006).

### QTL for repeated OF test trials

Because ethological tests for emotionality basically measure behavioral reaction toward novelty, it has been considered that repeated exposure to these tests measures a different aspect of behavior; prior experience of the same test causes adaptation to the situation (Broadhurst 1958), and therefore, behavior in the second trial reflects ‘habituation’ toward the novel environment to a greater extent (Bolivar *et al.* 2000) and memory of the previous session (Müller *et al.* 1994). QTL studies performed with repeated exposure of OF and LD revealed different QTL associated with the first trial and repeated trials (Gershenfeld & Paul 1997; Gershenfeld *et al.* 1997; Turri *et al.* 2001). In this study, OF tests were performed on two consecutive days, and consomic strains that showed significant differences from B6 were considered to have three different kinds of QTL: first-trial-specific QTL, second-trial-specific QTL and common QTL for both trials. We found that measurements related to stretch attend and central aversion tended to have a large effect from first-trial-specific QTL. Thus, stretch attend and indices for central aversion may particularly reflect response to novelty. Rearing contributes mainly to second-trial-specific QTL, and thus, it may be considered to be related to habituation or memory. The temporal changes in rearing support this idea (Takahashi *et al.* 2006; Vadasz *et al.* 1992), and this behavior has also been reported to have a relationship with the size of terminal mossy fiber projections to the hippocampus (Crusio 2001; Crusio *et al.* 1989a,b), which is closely involved in the processing of information about the environment (Schmajuk 1984) and exploratory learning (Moser *et al.* 1994). Examination of learning tasks in this consomic panel will give important insight into this result. It is hard to explain what kind of behavioral aspect contributes to the common QTL related to both trials. It may reflect aspects such as aversion toward the light, spontaneous activity or strong emotional reactivity that persists despite two exposures to the OF apparatus.

### Principal component analysis

To find the fundamental structure that underlies the anxiety-like behavior of the B6-ChrN^MSM^ consomic panel, we conducted multivariate analysis. Principal component analysis on phenotypic correlation extracted factors that were relatively test specific (Table S4). This result corresponds to the previously reported ‘instrument factors’ or ‘test session factors’ (Fernandes *et al.* 1999; Henderson *et al.* 2004; Royce *et al.* 1973). By using genetic correlations for the analysis, we were able to extract more cross-test factors compared to the principal component analysis of phenotypic correlations. However, there are some factors that have test-specific characteristics especially for OF and EPM. It has been reported that these two tests give contradictory results: the same mouse strain has been defined as ‘anxious’ with EPM and ‘nonanxious’ with OF (Rogers *et al.* 1999; Trullas & Skolnick 1993). Factor analyses of each OF and EPM with multiple measurements revealed a few inter-test correlations, strongly between locomotor activity factors in both tests, but many factors were independent between tests (Carola *et al.* 2002; Ramos and Mormède, 1998). Our results also supported this relationship, and only factor 1 had loadings from locomotor activity measurements of both tests. EPM open-arm exploration exclusively loaded on factor 3, while OF indices loaded the other factors. In contrast, LD test indices had close relations with some OF behaviors. LD transition and dark box preference loaded on factor 1 general locomotor activity, and latency to first transit loaded on factor 4, which has a large loading from OF stretch attend.

By calculating the factor scores in each consomic strain, we were able to map chromosomes associated with these five factors. The relationship between locomotor activity and autonomic reactivity in OF has long been discussed, with some findings of a strong negative correlation and others finding a positive, or no, correlation between those measurements (Archer 1973; Blizard *et al.* 2007; Takahashi *et al.* 2006). This result suggested there may be a moderate negative genetic correlation between factor 1 and factor 5. There may be not only a negative genetic correlation in some chromosomes (e.g. chromosomes 1, 6C, 11, 12C and 17) but also some independent genetic basis (e.g. chromosomes 2, 3, 6T, 9, 15, 16 and 19) or even reverse correlation (B6-Chr14^MSM^) between general locomotor activity and autonomic emotionality.

Numerous QTL have been reported for emotionality-related behaviors; almost all chromosomes (except 9, 13 and Y chromosomes) possess at least one QTL (for reviews, see Flint 2002; Willis-Owen & Flint 2006). We found that 13 of 17 chromosomes affect at least one of five emotionality-related factors; that is, our screening of consomic strains detected 58% of reported chromosomes and two new chromosomes, 9 and 13. Further analyses of consomic strains will reveal the relation between genetic loci of our consomic strains and other QTL studies and will provide deeper insight into the loci from psychological perspectives.

In this study, the use of B6-ChrN^MSM^ consomic panel mice allowed us to identify an extensive number of chromosomes related to anxiety-like behaviors because of the large genetic distance between B6 and MSM. These consomic strains showed substantially large effects on the phenotype, and are thus expected to be good tools for identifying the QTG (Hitzemann *et al.* 2003) and elucidate the genetic architecture of emotionality.

### Methodological issues

All animals in this study were singly housed for 1 week before the tests to measure home cage activity and kept individually until the end of screening. It has been reported that single housing changes some perspectives of an animal's behavior and the physical stress response (Brain 1975; Hilakivi *et al.* 1989; Stranahan *et al.* 2006). Therefore, this housing episode may have affected their anxiety-like behavior in our consomic mouse strains. In addition, behavioral tests were performed in a fixed order. Because all these ethological tests are related to novelty, previous experience of other novel situations and handling history affects an animal's behavior in subsequent ethological tests (McIlwain *et al.* 2001; Schmitt & Hiemke 1998); therefore, our factor structures derived from principal component analysis might have been affected by this test sequence. These effects of single housing and test sequence will be interesting to compare with other large datasets obtained from behavioral screening with high-throughput protocols for genetically modified mice in the future, as this comparison will aid a better understanding of environment–genetic interaction.

Finally, this study used almost the same number of animals for the host strain and each of the consomic strains. We could have achieved much larger statistical power of QTL detection if we had used a larger sample size for the host strain, at a ratio of 4.5:1, as suggested by Belknap (2003).

## Supplementary material

The following supplementary material is available for this article online from

http://www.blackwell-synergy.com/doi/full/10.1111/j.1601-183X.2008.00419.x

**Figure S1: Open-field behaviors of consomic mouse strains in the first trial.** Ambulation: distance traveled (cm); center time: time spent in the center squares; center ambulation: distance traveled within the center squares (cm); center %: ambulation/center ambulation; defecation: number of fecal boli; locomotion: walking and running around the arena; stretch attend: stretching the whole body forward while keeping the hind limbs in place; leaning: standing on the hind limbs with the forelimbs against the wall; rearing: standing on the hind limbs without touching the wall; grooming: licking and/or scratching the fur and licking the genitalia and tail; face washing: scrubbing the face with the forelimbs, not followed by grooming; and pausing: a brief moment of inactivity regardless of posture.

**Figure S2: Open-field behavior of consomic mouse strains in the second trial.** The same behavioral indices were observed as in trial 1.

**Figure S3: Behavior of consomic mouse strains in the LD box test.** LD transition: number of transitions between light and dark chambers; duration in dark box: time spent in the dark box; time to first transition: latency of first transition from light box to dark box.

**Figure S4: Behavior of consomic mouse strains in the EPM test.** Total distance (cm): distance traveled in the EPM; total arm entry: number of entries into both closed and open arms; closed-arm entry: number of entries into the closed arm; open-arm entry: number of entries into the open arm; open arm %: open-arm entry/closed-arm entry.

**Figure S5: Chromosomal mapping for five factors extracted from principal component analysis.** Factor 1: general locomotor activity; factor 2: thigmotaxis; factor 3: EPM open-arm exploration; factor 4: risk assessment; factor 5: autonomic emotionality. *Significant differences compared to B6 (*P* < 0.05 with a Bonferroni correction).

**Table S1:** Open-field behaviors.

**Table S2:** Effect of genotype, sex, and genotype–sex interaction on each behavioral variable.

**Table S3:** Phenotype and genetic correlations in the anxiety-like behaviors.

**Table S4:** Principal component analysis for anxiety-like behaviors using phenotype correlation.
