# Supplementary material for: Systematic analysis of emotionality in consomic mouse strains established from C57BL/6J and wild-derived MSM/Ms
Source: Genes Brain Behav. 2008 Nov;7(8):849–58. doi: 10.1111/j.1601-183X.2008.00419.x (PMC2667313; doi:10.1111/j.1601-183X.2008.00419.x)
Supplement: Supplementary file 1 [file gbb0007-0849-SD1.pdf]

Supplemental Figure 1. Open-field behaviors of consomic mouse strains in the first trial. Ambulation: distance traveled (cm). Center time: time spent in the center squares. Center amb: distance traveled within the center squares (cm). Center %: Ambulation/Center amb. Defecation: number of fecal boli. Locomotion: walking and running around the arena. Stretch-attend: stretching the whole body forward while keeping the hindlimbs in place. Leaning: standing on the hindlimbs with the forelimbs against the wall. Rearing: standing on the hindlimbs without touching the wall. Grooming: licking and/or scratching the fur, licking the genitalia and tail. Face-washing: scrubbing the face with the forelimbs, not followed by grooming. Pausing: a brief moment of inactivity regardless of posture.

Ambulation

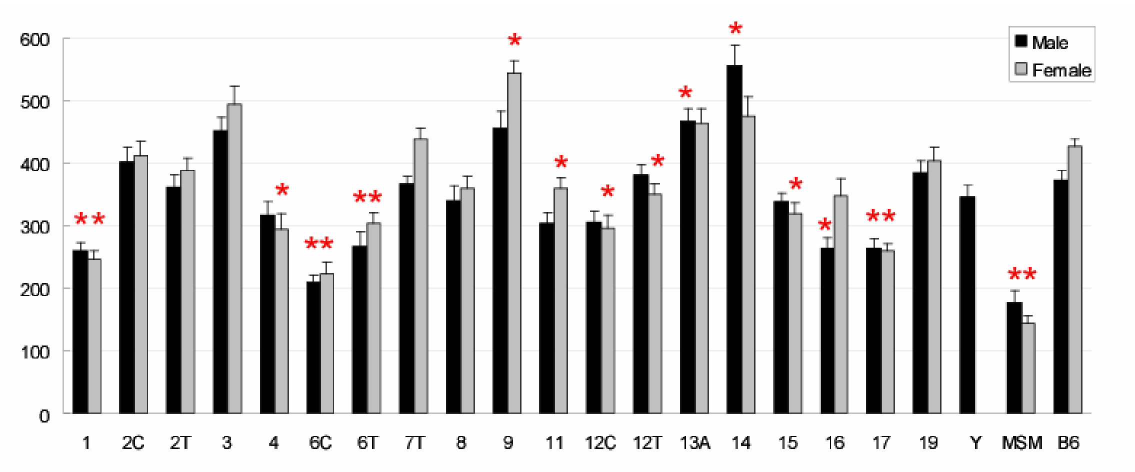

Center time

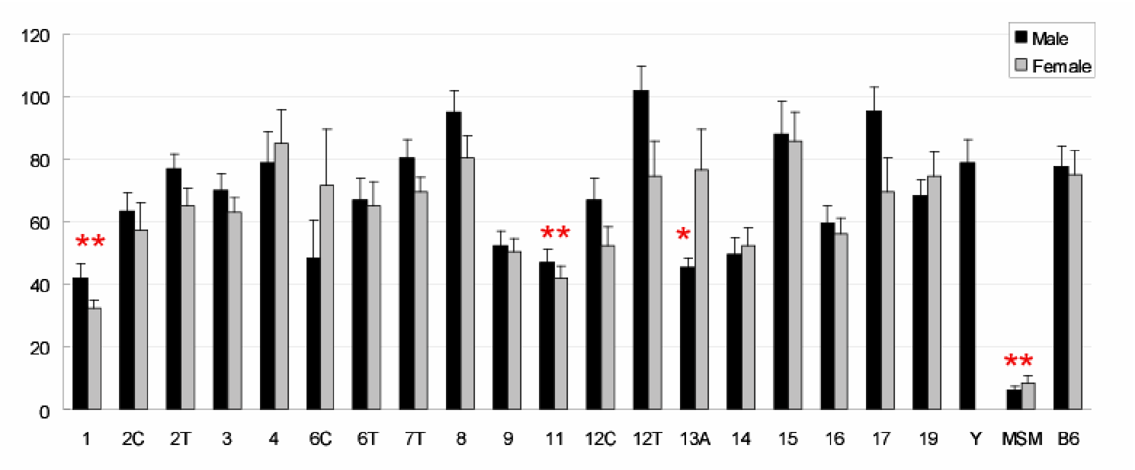

Center amb

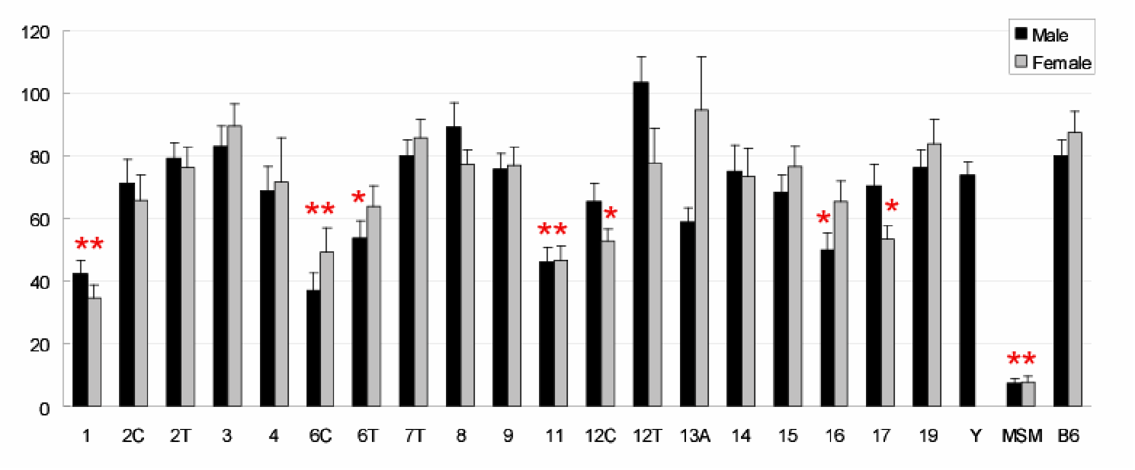

Center amb %

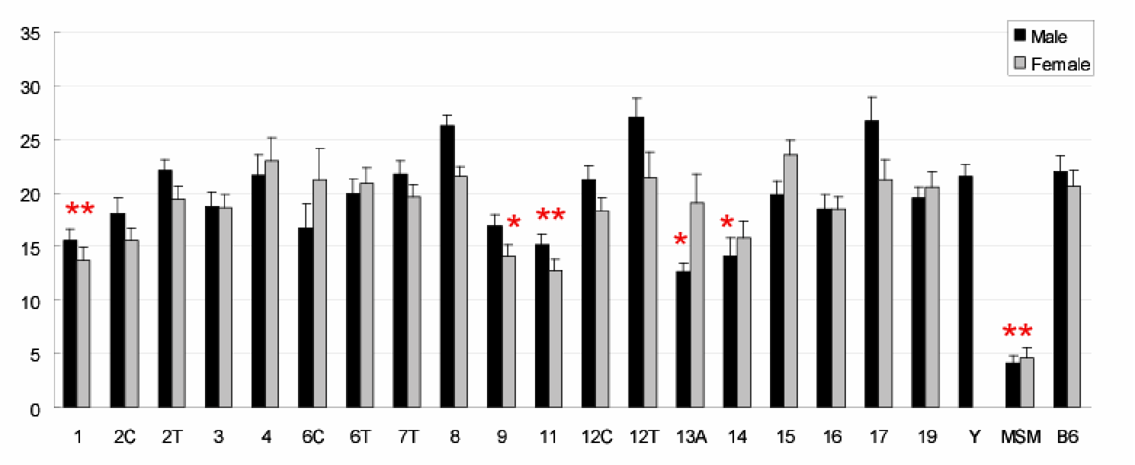

Defecation

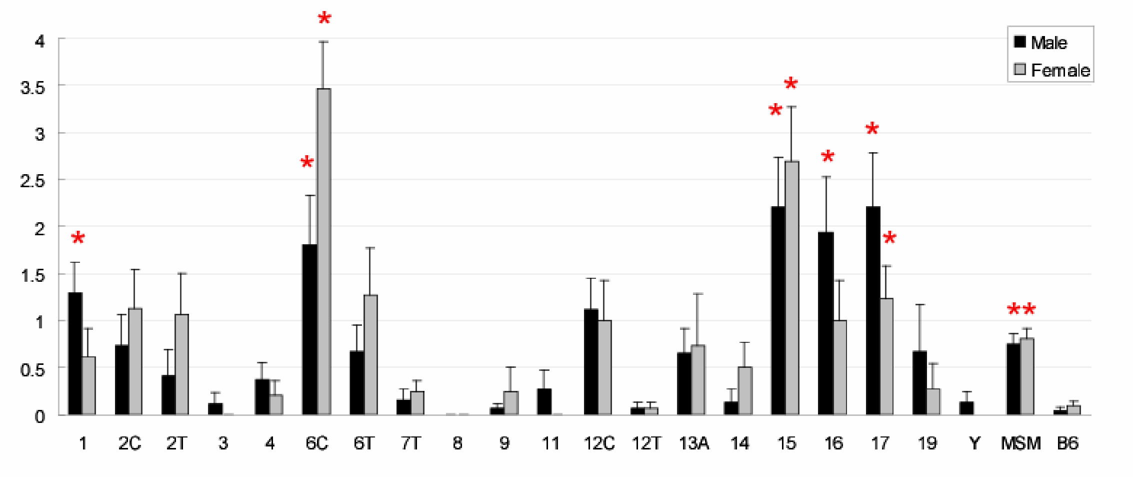

Locomotion

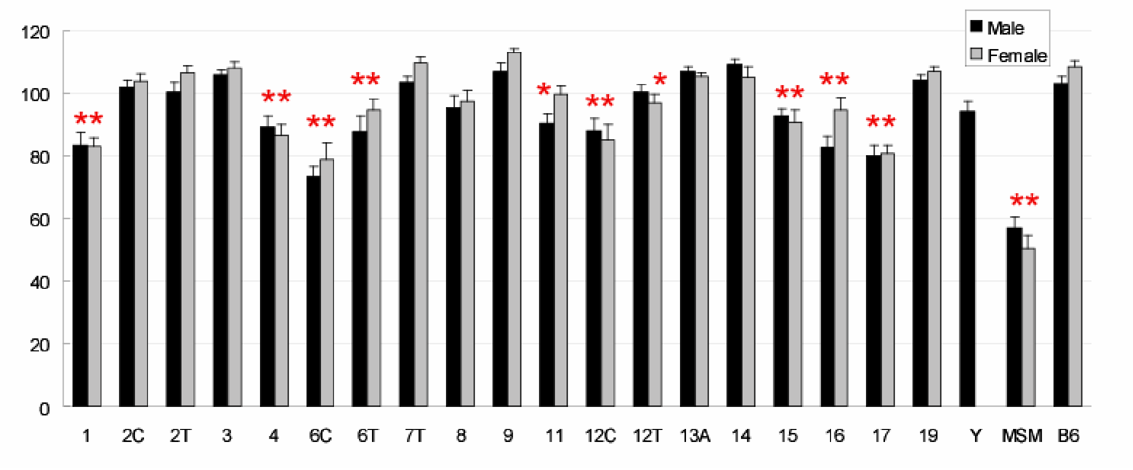

Stretching

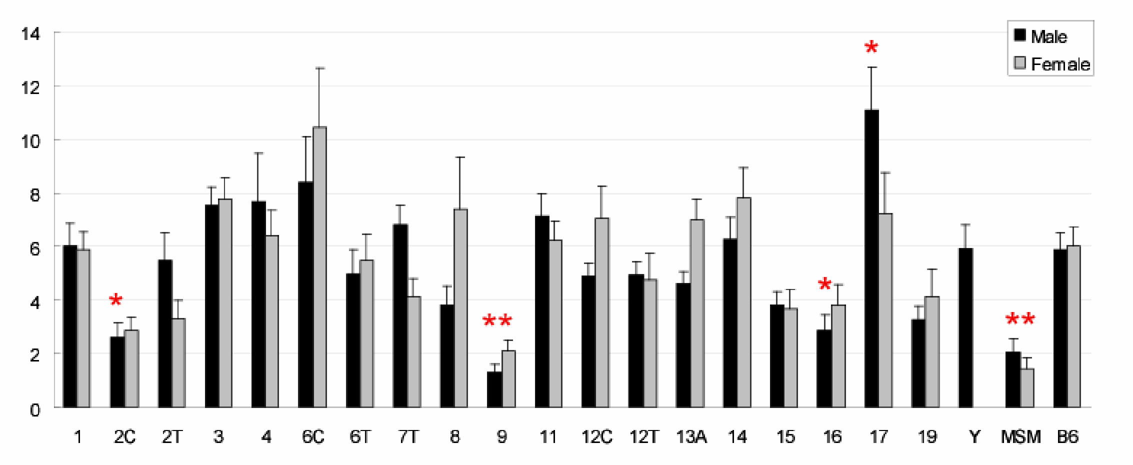

Leaning

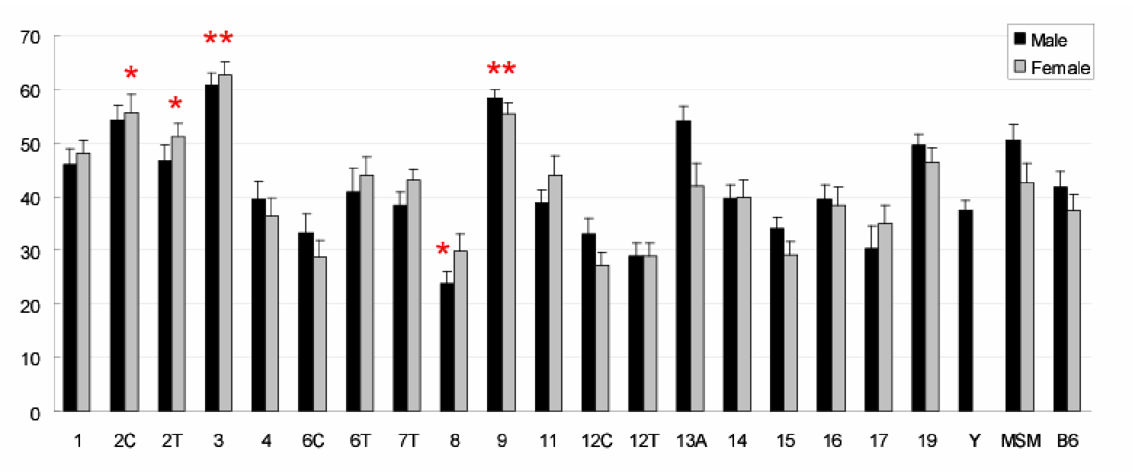

Rearing

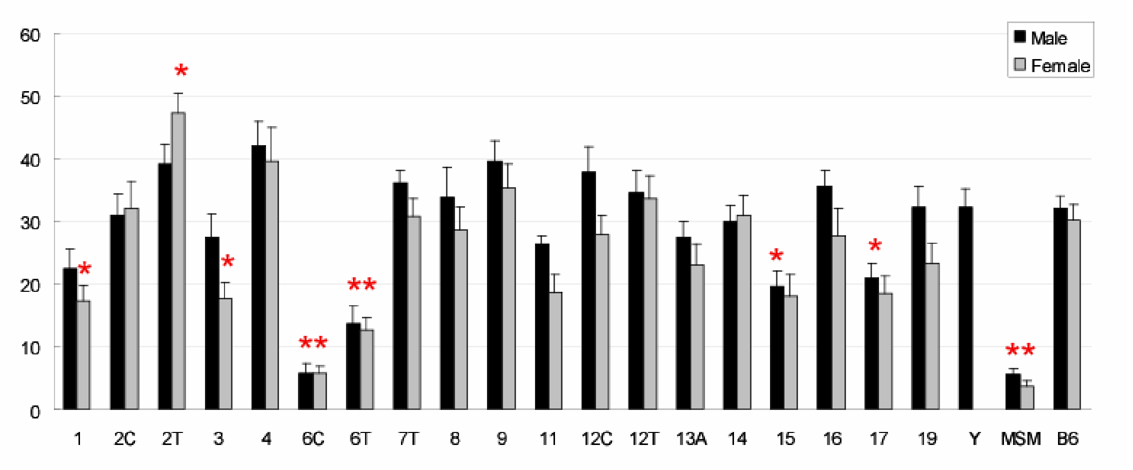

Grooming

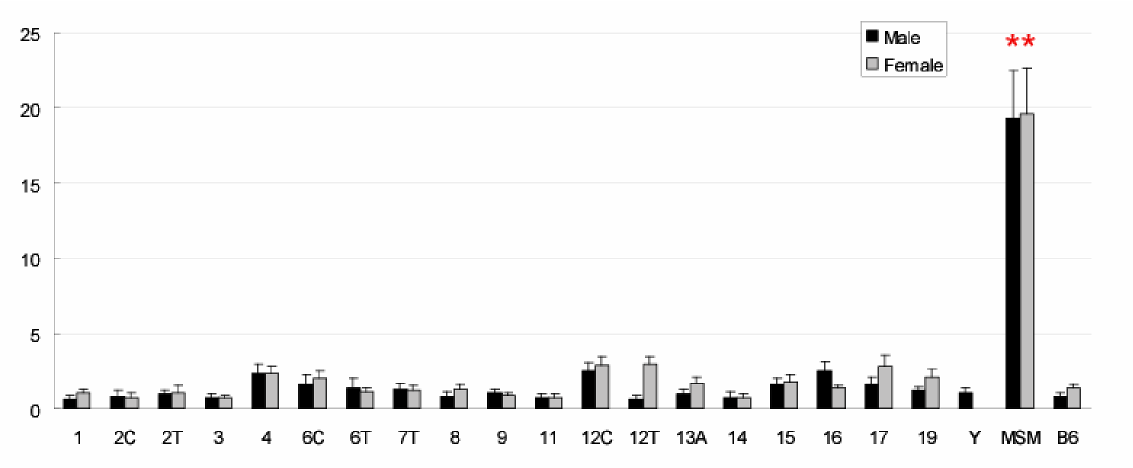

Face-washing

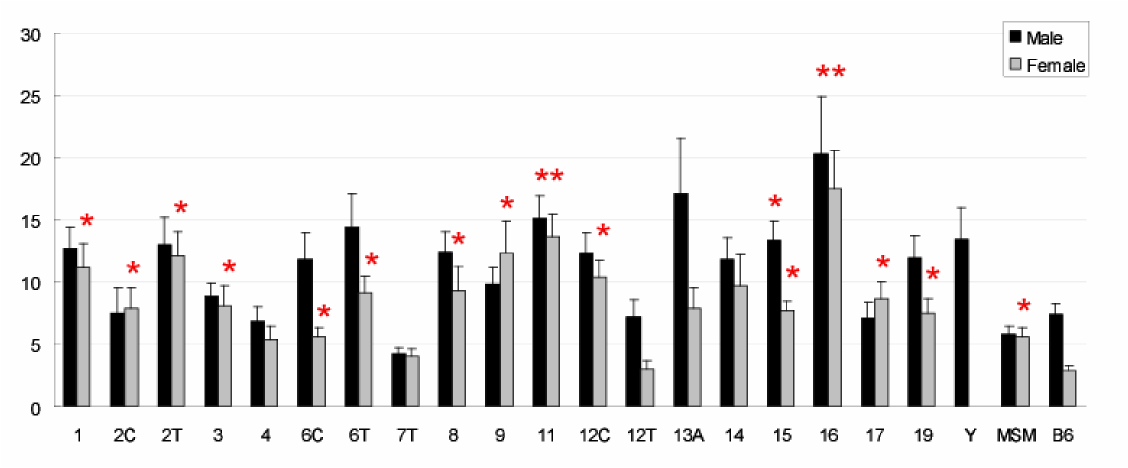

Jumping

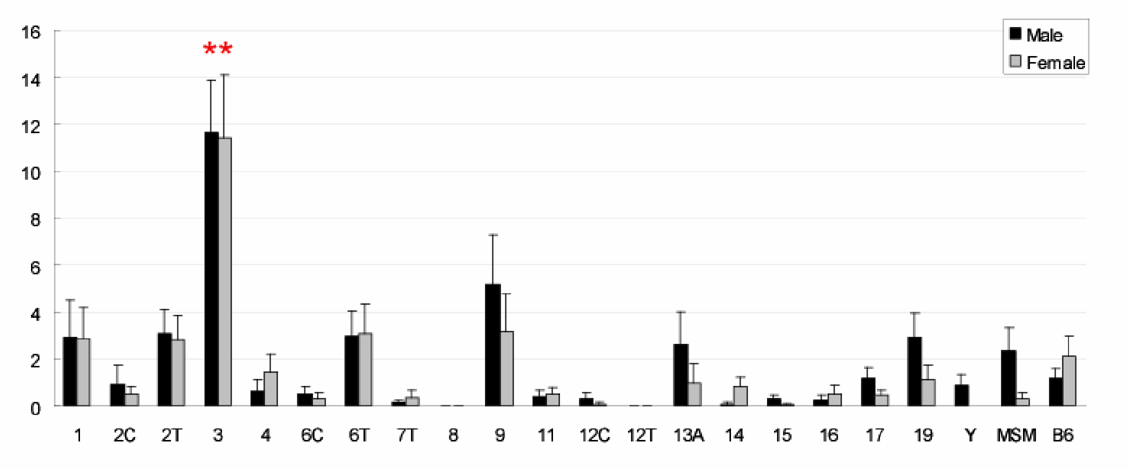

Pausing

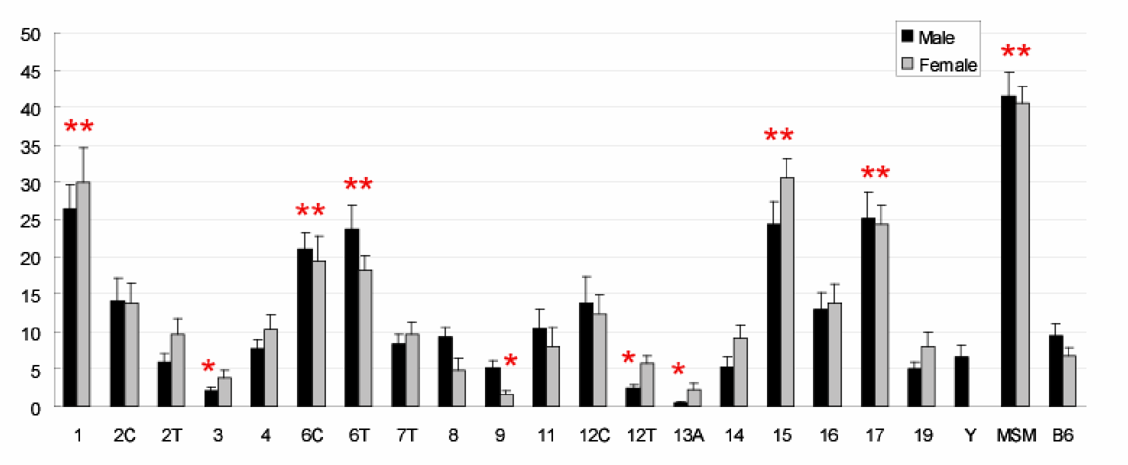

Supplemental Figure 1

Supplemental Figure 2. Open-field behavior of consomic mouse strains in the second trial. The same behavioral indices were observed as in trial 1.

Ambulation

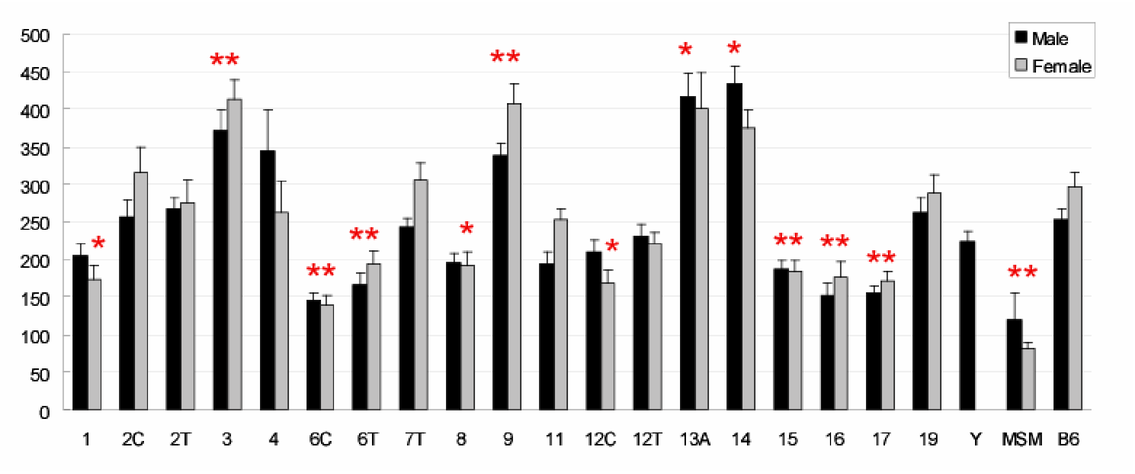

Center time

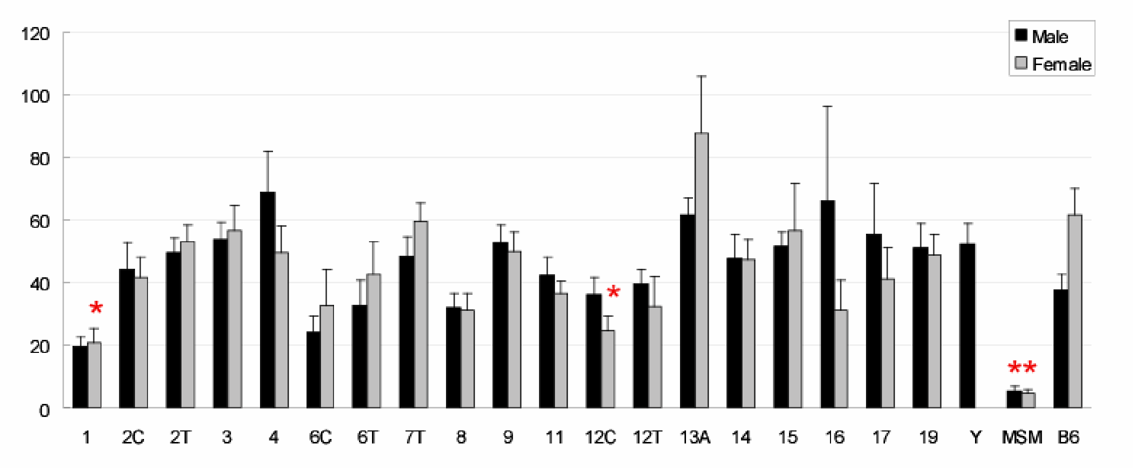

Center amb

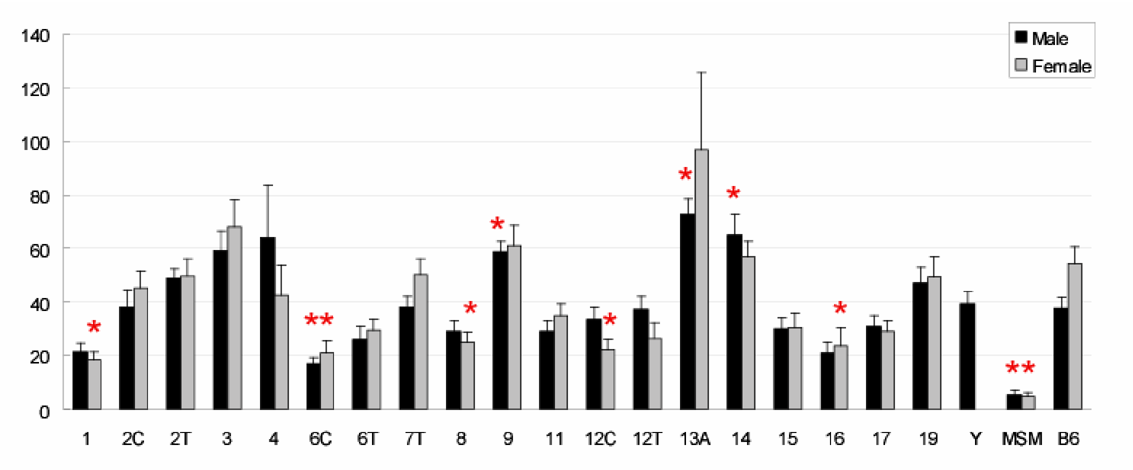

Center amb %

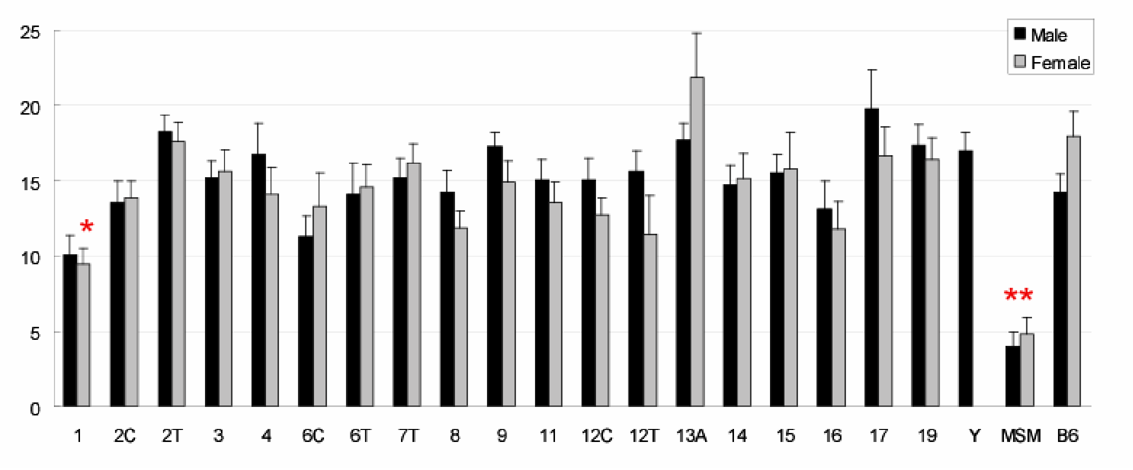

Defecation

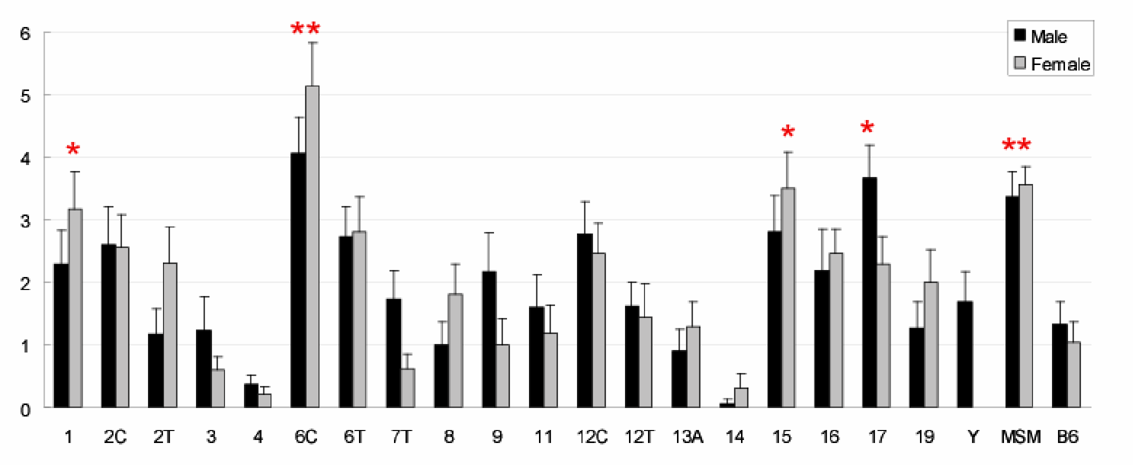

Locomotion

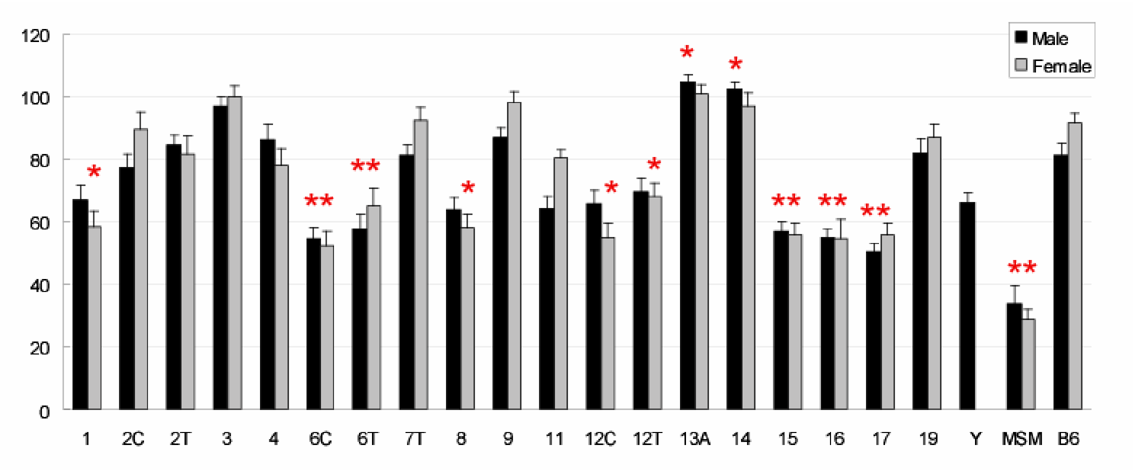

Stretching

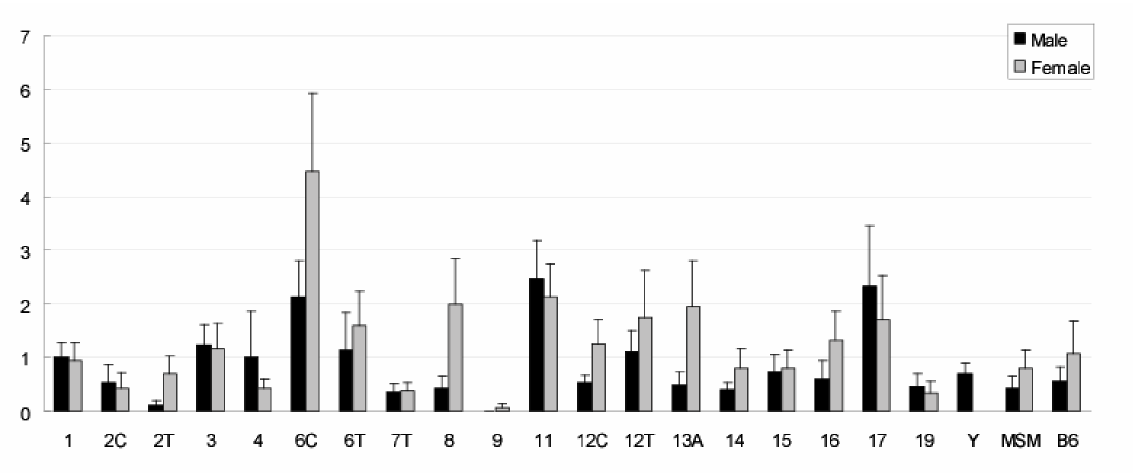

Leaning

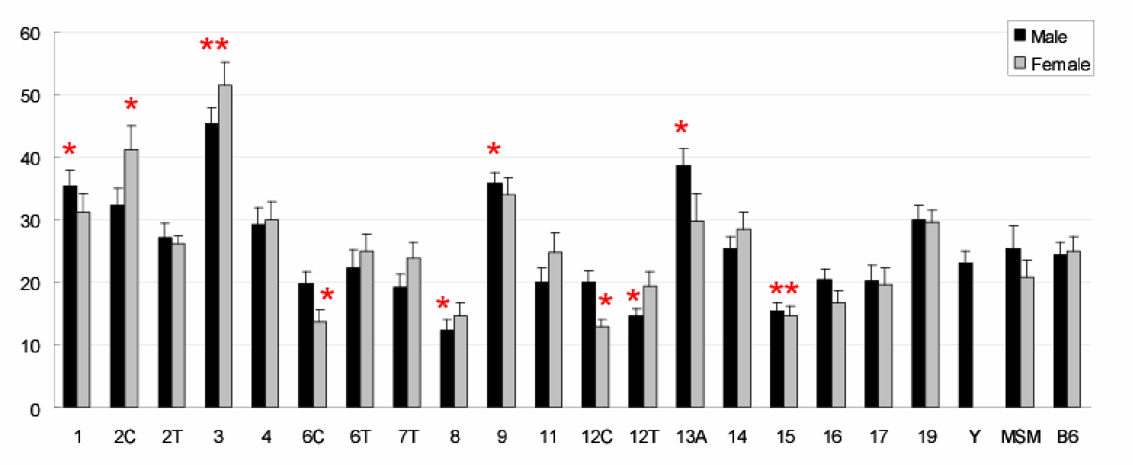

Rearing

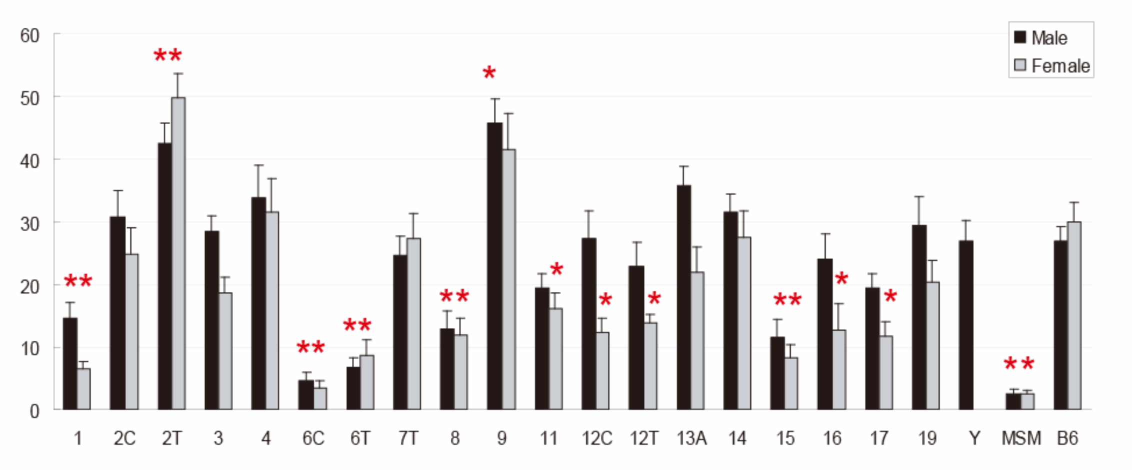

Grooming

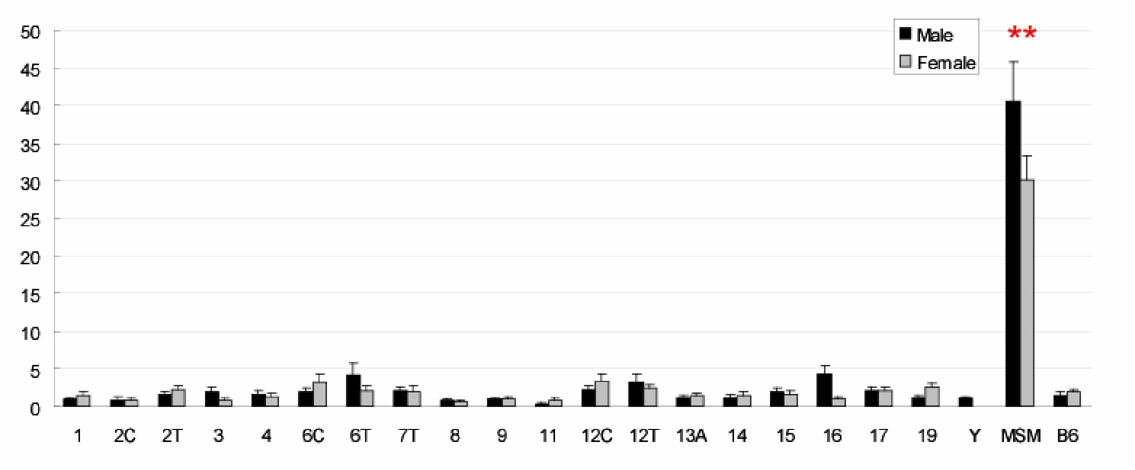

Face-washing

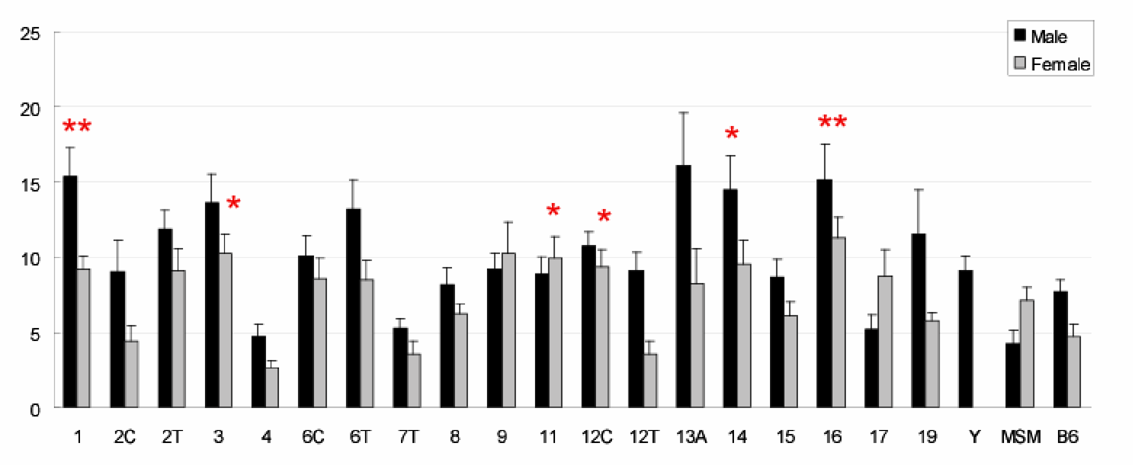

Jumping

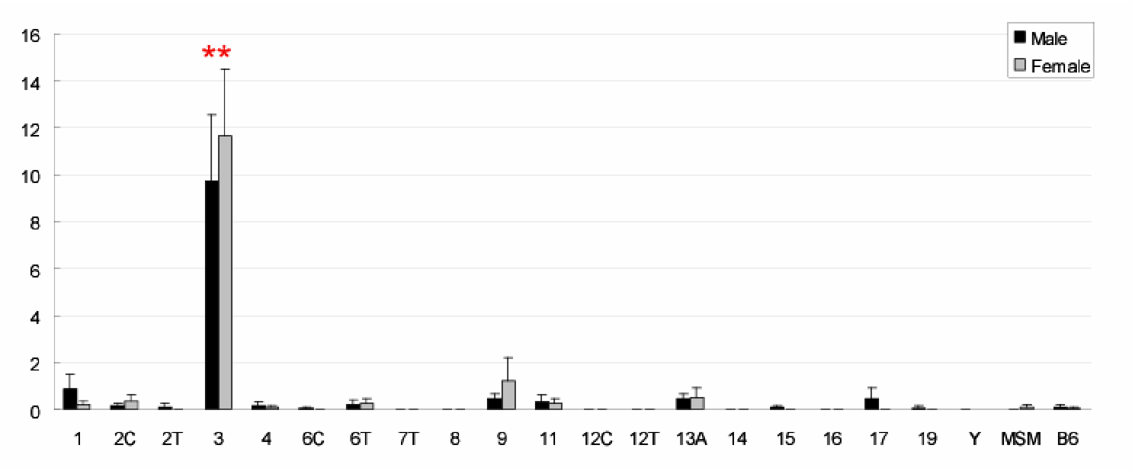

Pausing

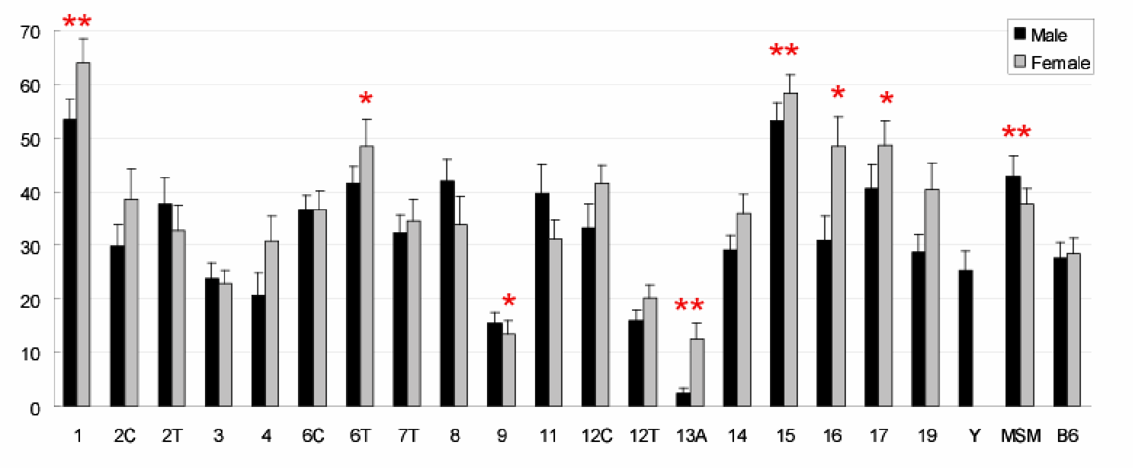

Supplemental Figure 2

Supplemental Figure 3. Behavior of consomic mouse strains in the light/dark box test. LD transition: number of transitions between light and dark chambers. Duration in dark box: time spent in the dark box. Time to 1st transition: latency of first transition from light box to dark box.

LD transition

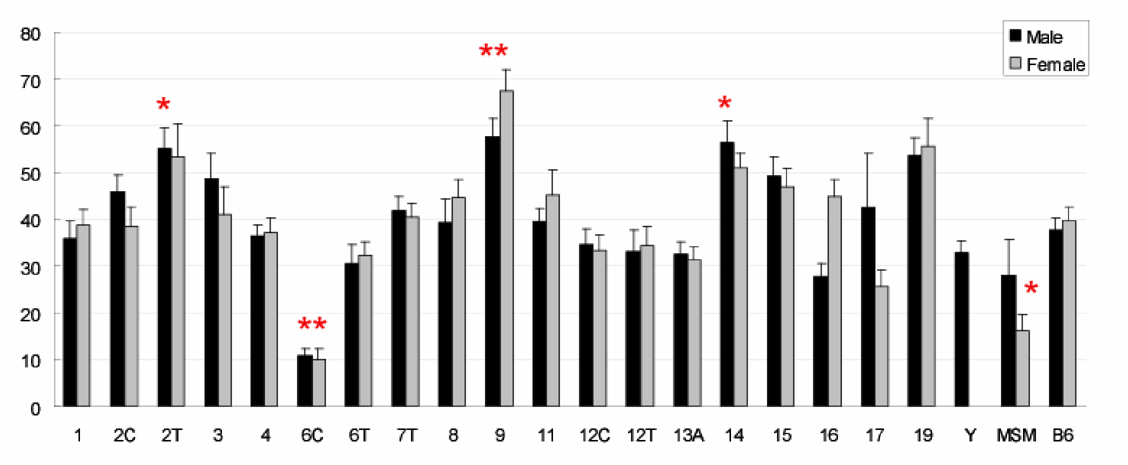

Duration in dark box

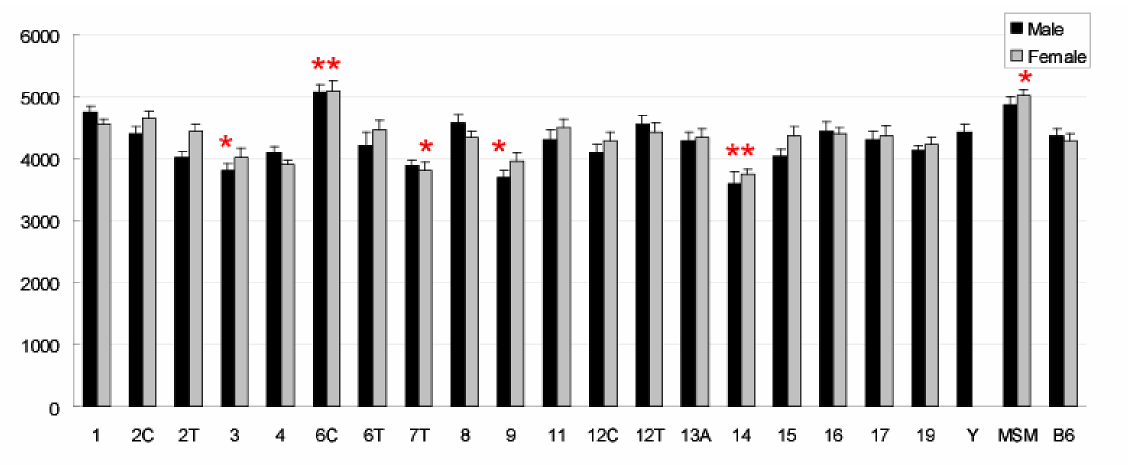

Time to 1st transition

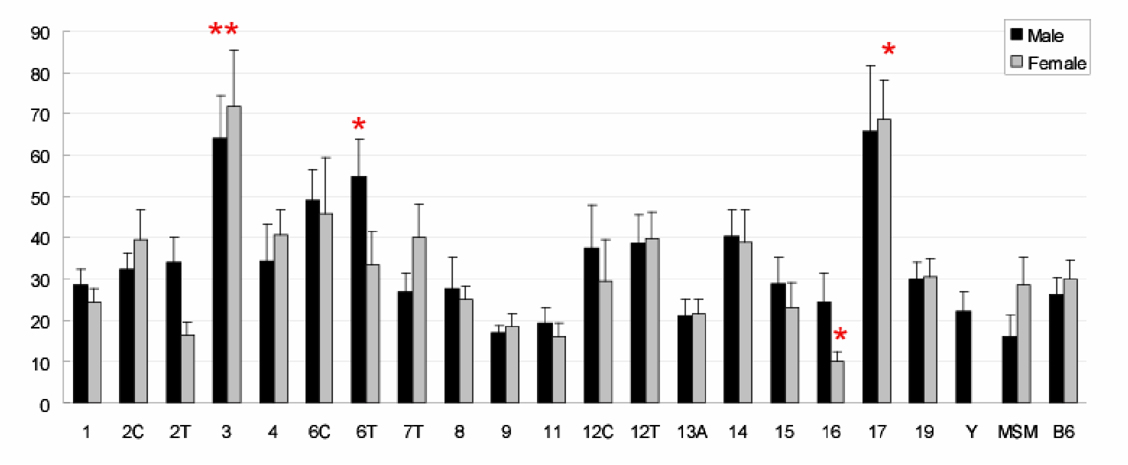

Supplemental Figure 3

Supplemental Figure 4. Behavior of consomic mouse strains in the elevated plus-maze test. Total distance (cm): distance traveled in the EPM. Total arm entry: number of entries into both closed and open arms. Closed-arm entry: number of entries into the closed arm. Open-arm entry: number of entries into the open arm. Open arm %: open arm entry/closed arm entry.

Total distance (cm)

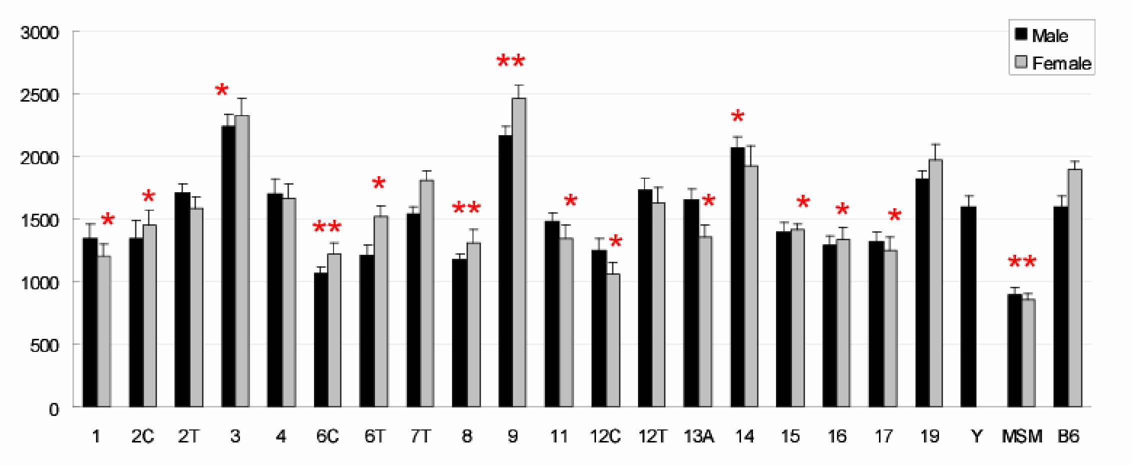

Total arm entry

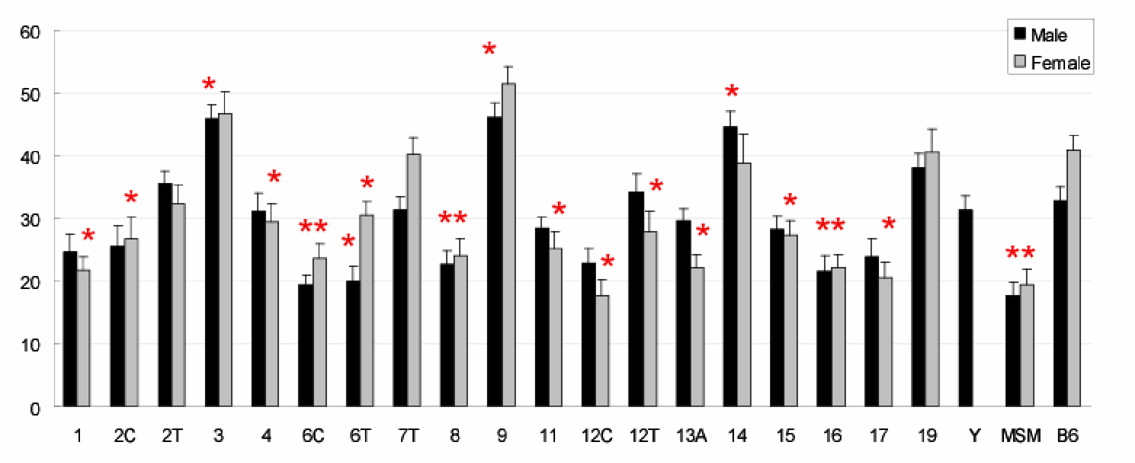

Closed-arm entry

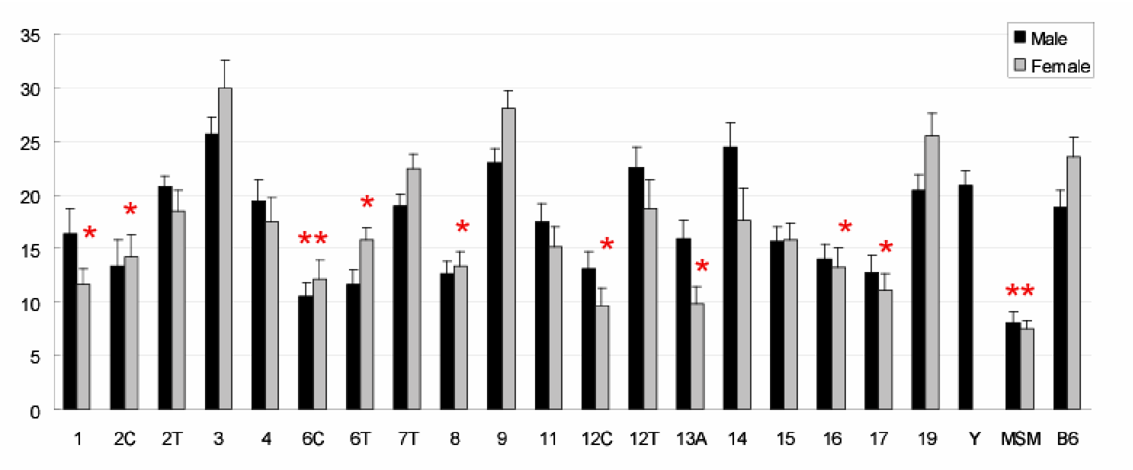

Open-arm entry

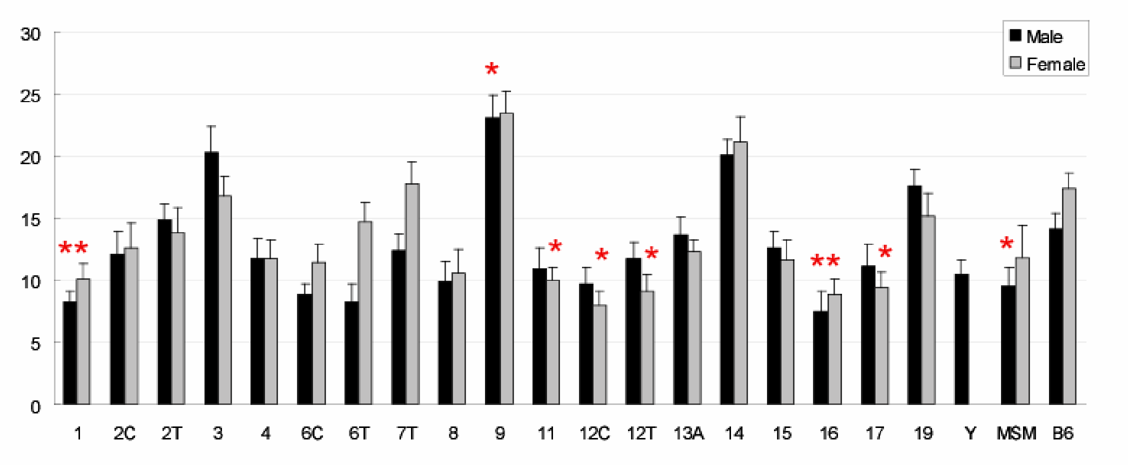

Open-arm %

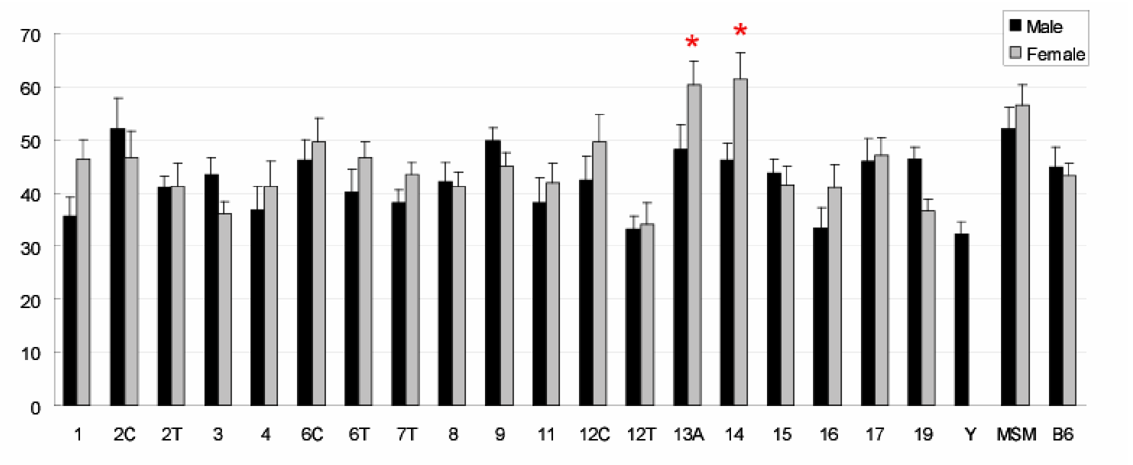

Open-arm time

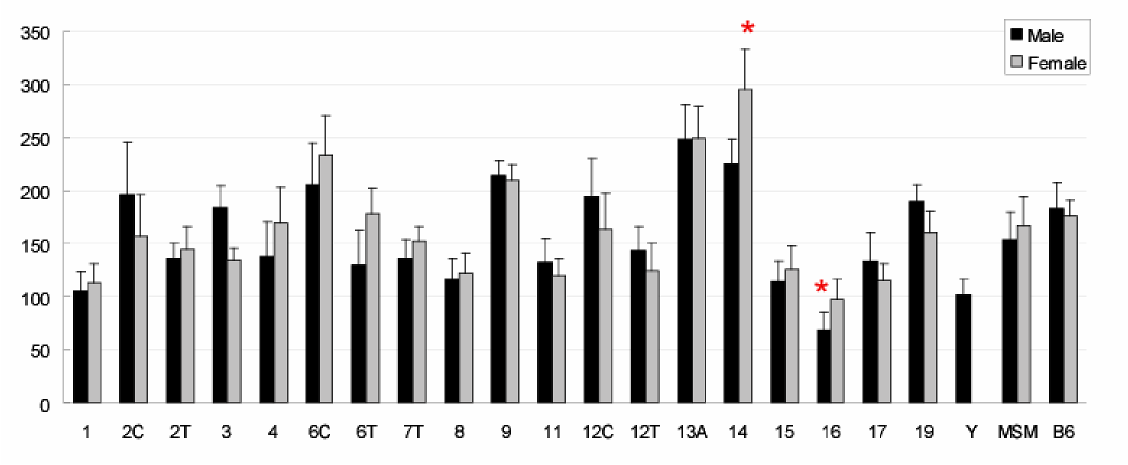

Supplemental Figure 4.
