# Supplementary material for: Systematic analysis of emotionality in consomic mouse strains established from C57BL/6J and wild-derived MSM/Ms
Source: Genes Brain Behav. 2008 Nov;7(8):849–58. doi: 10.1111/j.1601-183X.2008.00419.x (PMC2667313; doi:10.1111/j.1601-183X.2008.00419.x)

Supplemental Figure 5. Chromosomal mapping for five factors extracted from principal component analysis. Factor 1: General locomotor activity, Factor 2: Thigmotaxis, Factor 3: EPM open-arm exploration, Factor 4: Risk-assessment, Factor 5: Autonomic emotionality. * Significant differences compared to B6 (p<.05 with a Bonferroni correction).

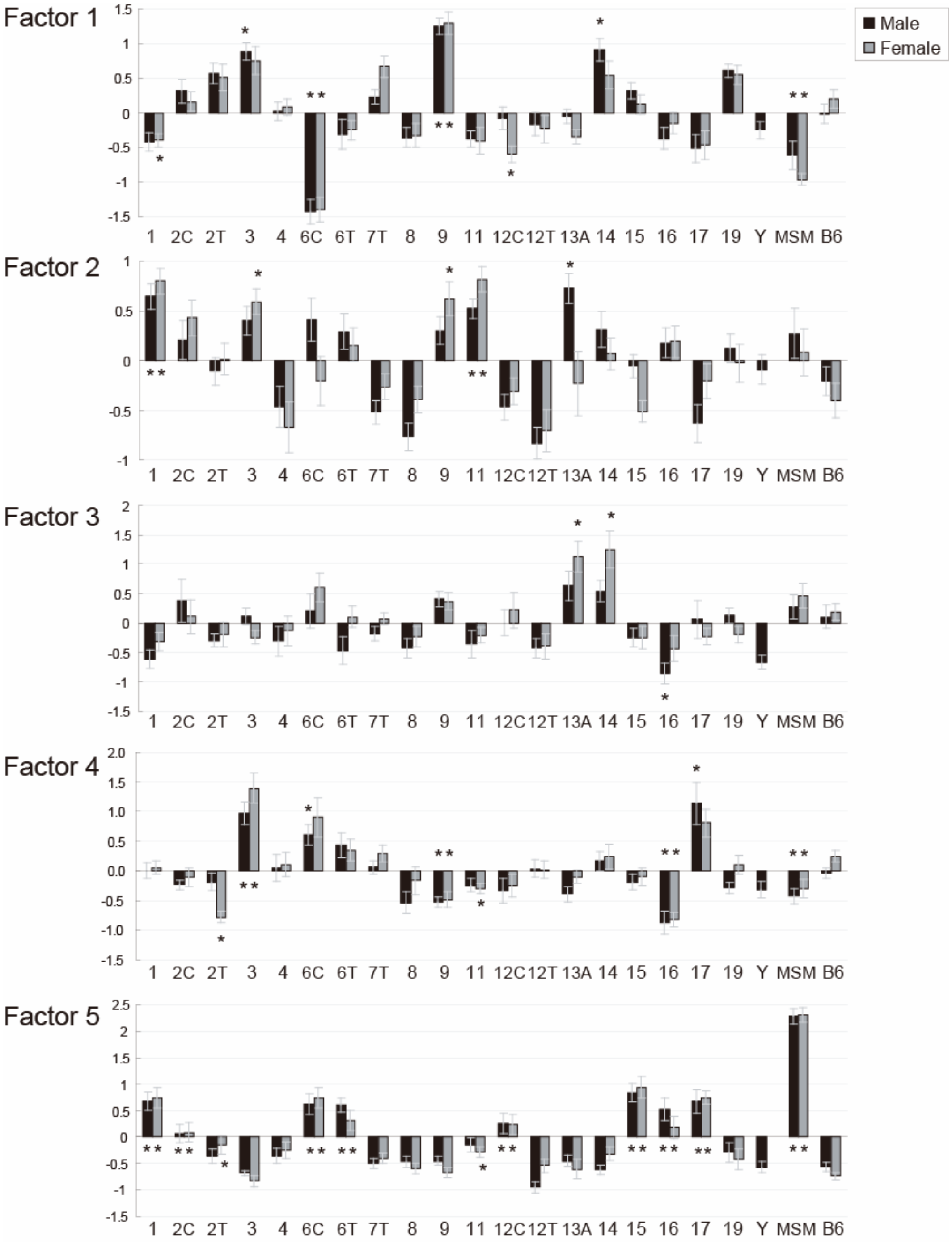

Supplement: Supplementary file 2 [file gbb0007-0849-SD2.pdf]
