# Supplementary material for: Systematic analysis of emotionality in consomic mouse strains established from C57BL/6J and wild-derived MSM/Ms
Source: Genes Brain Behav. 2008 Nov;7(8):849–58. doi: 10.1111/j.1601-183X.2008.00419.x (PMC2667313; doi:10.1111/j.1601-183X.2008.00419.x)
Supplement: Supplementary file 3 [file gbb0007-0849-SD3.pdf]

Supplemental Table 1. Open-field behaviors

Sniffing	Sniffing the arena and air, identified by characteristic movements of the nose and whiskers.
Locomotion	Walking and running around the arena.
Stretch attend posture (Stretch-attend)	Stretching the whole body forward while keeping the hindlimbs in place.
Leaning-against-wall (Leaning)	Standing on the hindlimbs with the forelimbs against the wall.
Rearing	Standing on the hindlimbs without touching the wall.
Grooming	Licking and/or scratching the fur, licking the genitalia and tail.
Face-washing	Scrubbing the face with the forelimbs, not followed by grooming.
Digging	Trying to dig a hole in the arena or the corner of the wall.
Gnawing	Gnawing mainly on the corner of the wall.
Jumping	Jumping vertically.
Pausing	A brief moment of inactivity regardless of posture.
Freezing	Stationary state lasting more than 3 seconds.
