# Supplementary material for: Systematic analysis of emotionality in consomic mouse strains established from C57BL/6J and wild-derived MSM/Ms
Source: Genes Brain Behav. 2008 Nov;7(8):849–58. doi: 10.1111/j.1601-183X.2008.00419.x (PMC2667313; doi:10.1111/j.1601-183X.2008.00419.x)
Supplement: Supplementary file 4 [file gbb0007-0849-SD4.pdf]

Supplemental Table 2. Effect of genotype, sex, and genotype×sex interaction on each behavioral variable.

	Eta squared estimates		
	SS_G/SS_{Total}	SS_{Sex}/SS_{Total}	SS_{GxS}/SS_{Total}
Open-field trial 1			
Ambulation	0.044	0.000	0.003
Center time	0.035	0.001	0.008
Central amb	0.035	0.000	0.007
Central amb %	0.022	0.000	0.006
Defecation	0.350	0.000	0.047
Locomotion	0.010	0.000	0.000
Stretch-attend	0.071	0.000	0.015
Leaning	0.045	0.000	0.003
Rearing	0.076	0.003	0.004
Grooming	0.065	0.004	0.022
Face-washing	0.068	0.009	0.011
Jumping	0.263	0.001	0.006
Pausing	0.233	0.000	0.008
Open-field trial 2			
Ambulation	0.081	0.000	0.005
Center time	0.040	0.000	0.013
Central amb	0.094	0.000	0.009
Central amb %	0.018	0.000	0.004
Defecation	0.128	0.000	0.016
Locomotion	0.041	0.000	0.002
Stretch-attend	0.086	0.006	0.021
Leaning	0.089	0.000	0.006
Rearing	0.139	0.006	0.010
Grooming	0.102	0.000	0.055
Face-washing	0.056	0.016	0.013
Jumping	0.408	0.000	0.005
Pausing	0.099	0.003	0.007
Light/dark box			
LD transition	0.056	0.000	0.005
Duration in dark box	0.005	0.000	0.000
Time to 1st transition	0.009	0.000	0.001
Elevated plus-maze			
total distance (cm)	0.777	0.002	0.059
Total arm entry	0.061	0.000	0.006
Closed-arm entry	0.059	0.000	0.009
Open-arm entry	0.069	0.000	0.007
Open-arm %	0.013	0.001	0.005
Open-arm time	0.055	0.000	0.005
