# Supplementary material for: Systematic analysis of emotionality in consomic mouse strains established from C57BL/6J and wild-derived MSM/Ms
Source: Genes Brain Behav. 2008 Nov;7(8):849–58. doi: 10.1111/j.1601-183X.2008.00419.x (PMC2667313; doi:10.1111/j.1601-183X.2008.00419.x)
Supplement: Supplementary file 5 [file gbb0007-0849-SD5.pdf]

Supplemental Table 3. Phenotype and genetic correlations in the anxiety-like behaviors

		Penotypic Correlation																					
		Open-Field													Light/dark box			Elevated plus-maze					
		AMB	CEN	CEN%	Time	Def	LO	ST	LE	RE	GR	FW	JP	PA	Transit	Dur.	Lat.	Dist.	Entry	O-ent	C-ent	O-%	O-Time
Genetic correlation: Open-field	Ambulation		0.573	-0.070	0.087	-0.314	0.827	-0.251	0.360	0.216	-0.235	-0.052	0.207	-0.560	0.370	-0.325	-0.071	0.545	0.531	0.438	0.458	0.056	0.170
	Center Amb.	0.730		0.723	0.710	-0.214	0.488	0.036	-0.125	0.209	-0.168	-0.209	0.088	-0.364	0.152	-0.175	0.032	0.273	0.294	0.239	0.258	0.057	0.109
	Center %	0.113	0.725		0.845	-0.015	-0.045	0.303	-0.460	0.108	-0.039	-0.219	-0.054	-0.033	-0.098	0.013	0.125	-0.052	-0.019	-0.037	0.009	0.057	0.049
	Center Time	0.229	0.796	0.959		-0.045	0.078	0.276	-0.444	0.154	-0.055	-0.253	-0.069	-0.108	-0.025	-0.063	0.089	0.005	0.049	0.004	0.086	0.100	0.105
	Defecation	-0.490	-0.340	0.081	0.034		-0.348	0.032	-0.100	-0.155	0.135	0.039	-0.040	0.333	-0.149	0.147	0.055	-0.210	-0.206	-0.199	-0.143	0.067	0.005
	Locomotion	0.923	0.827	0.345	0.422	-0.459		-0.317	0.437	0.260	-0.234	-0.019	0.169	-0.550	0.351	-0.275	-0.118	0.480	0.478	0.411	0.393	-0.028	0.093
	Stretching	-0.099	0.096	0.366	0.310	0.174	-0.051		-0.380	-0.194	-0.004	-0.178	-0.041	0.139	-0.241	0.100	0.179	-0.089	-0.080	-0.087	-0.044	0.101	0.113
	Leaning	0.428	-0.024	-0.485	-0.433	-0.305	0.337	-0.360		0.075	-0.121	0.179	0.414	-0.222	0.224	-0.142	-0.007	0.322	0.272	0.247	0.206	-0.039	0.021
	Rearing	0.521	0.618	0.412	0.427	-0.447	0.611	-0.192	0.078		-0.046	-0.059	0.018	-0.384	0.171	-0.213	-0.062	0.176	0.154	0.128	0.132	0.014	0.089
	Grooming	-0.524	-0.663	-0.634	-0.594	0.066	-0.725	-0.338	0.028	-0.469		0.034	-0.098	0.085	-0.083	0.048	-0.005	-0.151	-0.173	-0.152	-0.138	0.014	-0.048
	Face-wash	0.014	-0.184	-0.137	-0.183	0.110	0.032	-0.168	0.094	0.095	-0.262		0.020	-0.001	0.072	0.023	-0.117	-0.056	-0.071	-0.035	-0.090	-0.075	-0.101
	Jumping	0.319	0.152	-0.109	-0.109	-0.238	0.255	0.050	0.707	-0.038	-0.091	0.008		-0.155	0.127	-0.126	0.081	0.256	0.234	0.207	0.185	0.018	0.070
	Pausing	-0.785	-0.758	-0.371	-0.430	0.556	-0.836	-0.111	-0.171	-0.684	0.636	-0.095	-0.199		-0.147	0.227	0.053	-0.374	-0.341	-0.309	-0.262	0.011	-0.140
Light/dark	Transition	0.699	0.519	0.105	0.177	-0.392	0.694	-0.284	0.387	0.562	-0.386	0.133	0.250	-0.451		-0.462	-0.286	0.339	0.329	0.276	0.280	-0.028	-0.013
	Dark Duration	-0.693	-0.573	-0.225	-0.292	0.439	-0.651	-0.001	-0.250	-0.580	0.417	-0.016	-0.293	0.550	-0.714		-0.252	-0.298	-0.284	-0.214	-0.272	-0.033	-0.097
	Latency	-0.035	0.138	0.310	0.278	0.135	-0.067	0.539	-0.026	-0.231	-0.137	-0.297	0.376	0.045	-0.229	-0.123		-0.012	-0.018	-0.047	0.022	0.064	0.109
Elevated plus-maze	Total Distance	0.831	0.647	0.159	0.232	-0.461	0.777	-0.025	0.498	0.473	-0.452	-0.093	0.543	-0.693	0.674	-0.715	0.148		0.927	0.859	0.686	-0.143	0.093
	Total entry	0.796	0.596	0.093	0.173	-0.454	0.734	-0.088	0.502	0.416	-0.352	-0.151	0.519	-0.612	0.681	-0.683	0.104	0.973		0.873	0.806	-0.067	0.090
	Close-arm entry	0.731	0.639	0.222	0.285	-0.482	0.726	-0.035	0.413	0.466	-0.427	-0.112	0.497	-0.651	0.627	-0.627	0.128	0.949	0.947		0.414	-0.488	-0.230
	Open-arm entry	0.752	0.442	-0.091	-0.001	-0.344	0.626	-0.144	0.537	0.283	-0.195	-0.179	0.463	-0.462	0.641	-0.646	0.054	0.846	0.907	0.724		0.467	0.445
	Open-arm %	0.034	-0.262	-0.443	-0.402	0.172	-0.151	-0.027	0.156	-0.330	0.334	-0.179	-0.049	0.246	-0.086	0.057	-0.040	-0.189	-0.127	-0.419	0.277		0.758
	Open-arm Time	0.419	0.118	-0.218	-0.163	-0.024	0.260	0.107	0.225	-0.026	-0.022	-0.202	0.093	-0.264	0.055	-0.206	0.049	0.305	0.328	0.098	0.577	0.705	

High correlations over 0.9 are red.
