# Supplementary material for: Systematic analysis of emotionality in consomic mouse strains established from C57BL/6J and wild-derived MSM/Ms
Source: Genes Brain Behav. 2008 Nov;7(8):849–58. doi: 10.1111/j.1601-183X.2008.00419.x (PMC2667313; doi:10.1111/j.1601-183X.2008.00419.x)
Supplement: Supplementary file 6 [file gbb0007-0849-SD6.pdf]

Supplemental Table 4. Principal component analysis for anxiety-like behaviors by using phenotype correlation

	1	2	3	4	5
Open-field					
Ambulation	**0.86**	−0.07	0.09	0.02	−0.01
Center amb	**0.68**	**0.58**	0.03	−0.03	−0.04
Center %	0.24	**0.87**	−0.03	0.02	0.06
Defecation	**−0.58**	0.03	0.11	0.09	0.34
Stretching	−0.17	**0.59**	0.07	0.21	−0.13
Leaning	0.27	**−0.71**	0.01	0.16	0.07
Rearing	**0.50**	0.10	0.02	−0.03	0.21
Grooming	−0.34	−0.35	0.07	−0.18	−0.46
Face-washing	−0.17	−0.16	−0.07	−0.07	**0.76**
Pausing	**−0.74**	−0.09	−0.01	−0.02	−0.05
Light/dark box					
Transition	**0.52**	−0.20	0.00	−0.11	0.33
First transit latency	−0.19	0.06	−0.01	**0.91**	−0.12
Dark box duration	−0.40	0.14	−0.02	**−0.55**	−0.22
Elevated plus-maze					
Total arm entry	**0.68**	−0.17	0.00	0.15	−0.16
Open-arm entry %	−0.06	−0.02	**0.94**	−0.07	−0.02
Open-arm time	0.12	0.00	**0.93**	0.03	−0.02

Factor loadings over 0.5 are boldfaced.
